# CD34^+^ PI16^+^ fibroblast progenitors aggravate neointimal lesions of allograft arteries via CCL11/CCR3-PI3K/AKT pathway

**DOI:** 10.7150/thno.104650

**Published:** 2025-01-20

**Authors:** Xiaodong Xu, Pengwei Zhu, Han Wang, Kai Chen, Liang Liu, Luping Du, Liujun Jiang, Yanhua Hu, Xuhao Zhou, Bohuan Zhang, Xiangyuan Pu, Xiaosheng Hu, Qingbo Xu, Li Zhang, Weidong Li

**Affiliations:** 1 Department of Cardiology, the First Affiliated Hospital, Zhejiang University School of Medicine, Hangzhou, China.; 2 School of Engineering and Materials Science, Queen Mary University of London, United Kingdom.; 3 Department of Cardiology, The Second Affiliated Hospital of Nanchang University, Nanchang, China.; 4 Department of Cardiology, and Institute for Cardiovascular Development and Regenerative Medicine, Xinhua Hospital Affiliated to Shanghai Jiaotong University School of Medicine, Shanghai, China.; 5 Department of Cardiovascular Surgery, First Affiliated Hospital, School of Medicine, Zhejiang University, Hangzhou, Zhejiang, China.

**Keywords:** CD34+ PI16+ fibroblast progenitors, CCL11, smooth muscle cells, genetic lineage tracing, transplant arteriosclerosis

## Abstract

**Rationale:** Transplant-accelerated arteriosclerosis is a common complication that limits the long-term survival of organ transplant recipients. While previous studies have indicated the involvement of CD34^+^ stem/progenitor cells (SPCs) in this process, their heterogeneity and potential adverse effects remains incompletely understood.

**Methods:** To investigate the role of CD34^+^ SPCs in transplant arteriosclerosis, we used various genetically modified mouse models, including BALB/c, C57BL/6J, CD34-CreER^T2^, Rosa26-tdTomato, Rosa26-iDTR, CD34-Dre, PI16-CreER^T2^, and CAG-LSL-RSR-tdTomato-2A-DTR mice. Single-cell RNA sequencing (scRNA-seq), chemokine antibody microarrays, ELISA assays, and immunohistochemistry were employed to identify fibroblast progenitors and their interactions with smooth muscle cells. Furthermore, *in vivo* and *in vitro* experiments targeting the CCL11/CCR3-PI3K/AKT signaling pathway were conducted to assess its role in the pathogenesis of transplant arteriosclerosis.

**Results:** Single-cell RNA-seq and genetic lineage tracing revealed a subpopulation of fibroblast progenitors, characterized by high CD34 and PI16 expression, which differentiated into a distinct chemotactic fibroblast subset. Proteomic and scRNA analysis revealed that this CD34^+^ PI16^-^ subgroup released CCL11 (Eotaxin-1), which promoted intimal hyperplasia through the paracrine activation of smooth muscle cells. Binding of CCL11 to its receptor CCR3 activated the PI3K/AKT signaling pathway in smooth muscle cells, driving their proliferation and migration. *In vivo*, overexpression of CCL11 promoted neointimal hyperplasia, while neutralizing CCL11 or inhibiting CCR3 alleviated neointimal formation.

**Conclusions:** These findings identified CD34^+^ PI16^+^ fibroblast progenitors that differentiate into specific chemotactic fibroblasts, releasing chemokines pivotal for neointima formation, suggesting a therapeutic strategy targeting their chemotactic activity.

## Introduction

Organ transplantation is the most effective treatment for patients with organ failure; however, transplant arteriosclerosis is a significant complication affecting the long-term survival of organ transplant recipients. Unfortunately, there are currently no efficient methods for its prevention and treatment 1. Transplant arteriosclerosis is characterized by endothelial damage, immune-mediated inflammatory responses, accumulation of smooth muscle cells (SMCs), adventitial fibrosis, and concentric intimal hyperplasia, ultimately leading to post-transplant organ ischemia and loss of function 2-5. Recent evidence suggests that stem/progenitor cells may play a crucial role in lesions formation 6. For instance, Sca1^+^ vascular stem cells are responsible for generating SMCs, indicating their vital role in vascular remodeling 7. Gli1^+^ cells in the adventitia can differentiate into vascular SMCs and are responsible for neointimal thickening after acute vascular injury 8. Lack of ATF3 in endothelial cells (ECs) reduces EC proliferation of large vessels and impairs regenerative processes in mouse aortic injury 9. Previous research has revealed that CD34^+^ KLF4^+^ stromal stem cells directly promote endometrial regeneration 10. However, few studies have focused on progenitor cells during adventitial hyperplasia and fibrosis in the tunica externa of allografts.

Due to their complex development and origins, fibroblasts represent a highly heterogeneous cell type within the vascular wall 11, 12. Under normal development of blood vessels, fibroblasts are sparsely distributed in the adventitia. However, upon vascular injury or inflammation, the adventitia exhibits an increase in specialized subpopulations of fibroblasts 13-15. Furthermore, fibroblasts, serving as vascular mesenchymal cells, provide essential niches and positional information for neighboring cells through the production of extracellular matrix and signaling molecules 16-18. While research on the mechanisms by which fibroblast progenitor cells participate in vascular remodeling is limited, single-cell RNA sequencing analysis have identified unique fibroblast progenitors, such as PI16 and SFRP2, that may differentiate into distinct fibroblast subpopulations 12, 19. Myofibroblasts, a distinct subgroup of fibroblasts, primarily secrete extracellular matrix and are closely associated with scar formation 20. Resident, but not circulating, Gli1^+^ cells expand and differentiate into myofibroblasts after kidney or heart injury 21. Another study indicated that PI16^+^ reticular cells in human palatine tonsils play a role in regulatory T cell activity 22. However, the biological origins and functions of specialized fibroblast subpopulations warrant further comprehensive investigation.

Not all activated fibroblasts or mesenchymal stem cells function as fibroblast progenitors. Hu and colleagues identified that the adventitia gathers abundant stem cells in vein grafts, including Sca1^+^, cKit^+^, CD34^+^, and Flk1^+^ cells 23-25. Subsequently, Jiang *et al.* found that resident CD34^+^ cells can repair ECs after vessel injury, while the CD34^+^ cells derived from bone marrow can evolve into inflammatory cells. Additionally, the potential significant influence of CD34^+^ cells on neointima formation is of considerable importance 26. CD34^+^ cells are predominantly distributed in the adventitia, making it uncertain whether they can act as progenitors for fibroblasts and manipulate neointima formation. Fibroblasts are non-hematopoietic structural cells that determine the structure of organs, support the homeostasis of tissue-resident cells, and play a crucial role in fibrosis, cancer, autoimmunity, and wound healing 27. In addition, Matthew B. Buechler *et al.* found that the mouse COL15^+^ PI16^+^ cell population can act as a reservoir that can generate specialized fibroblasts in a wide range of homeostatic tissues and generate activated fibroblasts in diseases. Under perturbed conditions, these universal fibroblast subpopulations maintain the highest expression of stemness-related genes (CD34 and Ly6a) 12. This study utilized a dual recombinase-mediated lineage tracer mouse, CD34 Dre-rox and PI16 Cre-loxp, to fate-map CD34^+^ and PI16^+^ cells in artery homeostasis and after allograft. We found that CD34^+^ PI16^+^ cells generate functional fibroblasts in the vessel walls that release chemokine CCL11. This cytokine binds to the CCR3 receptor on SMCs and activates the intracellular PI3K-AKT signaling pathway, ultimately promoting SMC proliferation and migration. Neutralizing CCL11 or knocking down its most specific receptor, CCR3 significantly diminished local inflammation and neointimal thickness, indicating a novel potential therapeutic target for the treatment of transplant arteriosclerosis.

## Methods

### Animals

All animal procedures were conducted following the Guide for Care and Use of Laboratory Animals (8^th^ edition, 2011) published by the US National Institute of Health and were approved by the Institutional Animal Care and Use Committee of Zhejiang University School of Medicine. Both male and female mice were used in the experiments, and details of the mouse strains are provided in the Supplementary materials. Euthanasia was performed using carbon dioxide following the National Institutes of Health animal euthanasia guidelines and was approved by the local animal welfare committee.

### Establishment of mouse transplant arteriosclerosis model

In preparation for surgery, the Balbc donor mouse was anesthetized, and the thoracic aorta was collected. Subsequently adventitial adipose tissue was removed and soaked in heparinized saline (200UI/ml). The C57 mouse was anesthetized with isoflurane through a transparent sealed container (induction vaporizer at 3% and maintained at 1%-2%). Orthotopic carotid artery transplantation with end-to-end anastomoses was performed according to our previously described cuff-tail technique 23. Transplant recipient mice were fed increased jelly-containing NSAIDs during the perioperative period. For detailed surgical methods, see Supplementary materials.

### Primary cell extraction and magnetic bead sorting

The adventitial adipose tissue was carefully removed from the grafts, cut into 1-2 mm pieces with sterile scissors, and immersed in DMEM supplemented with 20% fetal bovine serum (FBS) for 20 min. These tissue fragments were then adhered to a T25 culture flask pre-coated with a suitable substrate. Subsequently, these fragments underwent incubation at 37°C in a 5% CO_2_ atmosphere for 2-4 h before the introduction of a complete growth medium composed of DMEM, 20% FBS, penicillin-streptomycin (1×), 50 nM beta-mercaptoethanol, and 10 ng/mL mouse leukemia inhibitory factor. The cells were allowed to migrate from the tissue and fill up the T25 culture flask, followed by 3-5 passages. Cell dissociation was carried out using 0.25% trypsin-EDTA. Subsequently, CD34-beads antibody (130-117-775, Miltenyi Biotec) with magnetic beads were used for cell staining. The CD34 positive and negative cells were screened and separated using the magnetic column with the help of magnetic attraction on the magnetic shelf.

### Chemokine antibody microarrays detection of cell supernatants

The CD34-positive and negative cells in good growth status were planted in 6-well plates. After serum-free culture for 12 h, the cell culture fluid was collected and centrifuged (2000 r/min) at 4 °C for 15 min. Furthermore, the target cell supernatant was collected for detection. Mouse chemokine array (QAM-CHE-1, RayBiotech) testing was performed on cell supernatant from CD34-positive and negative cell samples on admission according to the instructions provided by the manufacturer. Each cell supernatant sample was hybridized to the array overnight at 4 °C. All slides were scanned using a GenePix 4000 B microarray scanner and analyzed using GenePix Pro software (version 6.0). Protein levels were normalized to internal controls. Detailed surgical methods are demonstrated in Supplementary materials.

### Flow cytometric sorting and single-cell RNA sequencing

Four weeks after the operation, allografts from each experimental group were harvested from six male and female CD34CreER^T2^: Rosa26-tdTomato mice. Grafts were rinsed three times in phosphate-buffered saline (PBS), minced, and enzymatically dissociated in a shaking water bath at 37 °C with a digestion cocktail comprising papain, collagenase I, and dispase II as delineated in prior protocols. Cells liberated into the supernatant were harvested and suspended in DMEM with 10% FBS. Cells from each graft were pooled following digestion. Following adequate dissociation, the cell suspension was filtered through a 40-μm mesh to remove undigested debris and then centrifuged for 8 min at 500 g at 4 °C. Single cells were collected, washed with PBS, and resuspended in 0.04% BSA. Single mononuclear live cells positive for tdTomato (Hoechst positive and dead cell stain negative) were sorted using the BD FACS ARIA II flow cytometer (BD Biosciences). The sorted cells were then subjected to scRNA-seq. Libraries were prepared using the Chromium TM Single Cell 3' Reagent Kit v3 chemistry (10× Genomics) following the standard protocol. Libraries were sequenced on the Novaseq6000 PE150 platform (Illumina) with a paired-end 150 bp sequencing strategy. The 10× Chromium TM processes, library generation, and sequencing were performed by Novogene Co., Ltd (Beijing, China).

### Bone marrow transplantation

A lethal dose of whole-body X-ray irradiation (9.0 Gy) was administered to recipient mice. Subsequently, we harvested the bone marrow cells by collecting and flushing the cavities of femurs, as well as tibias isolated from donor mice using 1 mL RPMI 1640 medium (BI, 01-100-1A). Bone marrow cells were then passed through 40-μm cell strainers (Falcon, 352340) to obtain single cell suspensions, followed by re-suspension in RPMI 1640 medium before transplantation. After 6 h, irradiated recipient mice were administered with 5*10^6^ donor bone marrow cells via tail vein injection to form chimeric mice.

### Whole-mount staining

For 3D visualization, tissues were fixed overnight at 4 °C and washed thrice for 1 h in PBS. Samples were then treated with Cubic-L solution [composed of 10% (wt/wt) N-butyldiethanolamine (TCIchemicals, B0725) and 10% (wt/wt) Triton X-100 in distilled water] at 37 °C for two days. Subsequently, tissues were washed three times for 1 h in PBS, incubated in primary antibody solution for two days at 4 °C, and washed three times in PBS. The secondary antibody was incubated for one day at room temperature and then washed with PBS. The tissue was then cleared using Cubic-R+ solution [45% (wt/wt) antipyrine (TCIchemicals, D1876), 30% (wt/wt) nicotinamide (TCIchemicals, N0078), and 0.5% (vol/vol) N-butyldiethanolamine in distilled water]. Imaging was performed using a Leica TCSSP8 DIVE confocal microscope, and reconstructions were created using Leica's native LAS X software (version 4.5).

### Immunofluorescence

For immunofluorescence analysis, normal and allograft arterial tissues were procured and initially rinsed in PBS, followed by fixation in 4% paraformaldehyde at 4 °C for 2 h. Subsequently, the tissues underwent dehydration in a 30% sucrose solution at 4 °C overnight until thorough penetration was achieved. The specimens were then encased in optimum cutting temperature compound (O.C.T., Sakura, 4583) and cryopreserved at -80 °C or sectioned into 5-μm slices using a Cryostat (Leica CM1950). Sections were left to air-dry for approximately 30 min at ambient temperature, then blocked and permeabilized using 5% donkey serum (Solarbio®, SL050) with 0.1% Triton X-100 in PBS for 1 h. This was succeeded by primary antibody application overnight at 4 °C and subsequent incubation with Alexa Fluor-tagged secondary antibodies (Invitrogen, at 1:500 dilution) for 1 h. Nuclei were counterstained with DAPI (Servicebio, G1012), and sections were mounted with an anti-fade medium (Servicebio, G1401). Utilized primary antibodies included those against tdTomato (Rockland, 600-401-379; SICGEN, AB8181-200), GFP (Abcam, ab6662), CD34 (Abcam, ab81289), CD31 (R&D, AF3628), SMA-FITC (Sigma, F3777), CD45 (R&D systems, AF114), Ki-67 (Abcam, ab16667), PDGFRa (R&D systems, AF1062), Postn (R&D systems, AF2955), Vimentin (Abcam, ab8978), PI16 (R&D systems, AF4929), CCL11 (R&D systems, AF-420-NA), and CCR3 (Proteintech, 22351-1-AP). Secondary antibodies included donkey anti-rabbit IgG Alexa Fluor 555 (Invitrogen, A-31572), donkey anti-rabbit IgG Alexa Fluor 647 (Invitrogen, A-32795), donkey anti-rat IgG Alexa Fluor 647 (Invitrogen, A-48272), donkey anti-mouse IgG Alexa Fluor 488 (Invitrogen, A-21202), donkey anti-goat IgG Alexa Fluor 555 (Invitrogen, A-32816), and donkey anti-goat IgG Alexa Fluor 647 (Invitrogen, A-21447). For quality control, isotype control antibodies (Invitrogen) and secondary-only controls were employed to ensure antibody specificity and minimize non-specific background staining.

### SiRNA transfection

Pre-designed siRNA and negative control siRNA were procured from General Bio. SMCs were transfected with siRNA using Lipofectamine RNAiMAX reagent (Invitrogen, 13778150) in OptiMEM™ I Low Serum Medium (GIBCO, 31985070) according to the instructions by the manufacturer. After incubation at 37 °C for 6 h, the medium was replaced to complete the transfection for the subsequent experiment.

### *In vivo* expression of CCL11 in allografts

Adeno-associated virus (AAV) overexpression plasmid of CCL11 and negative control was constructed from OBiO Tech, Inc. (H12049, H24912). AAV fractions were first prepared by mixing 100 μL AAV (4*10^12^ VG/mL) with 100 μL 25% Pluronic F-127 (PF-127) gel for 30 min at 4 °C. After the aortic segments of BALB/c mice were implanted into C57BL/6J mice, the overexpression plasmids and vectors of the control and experimental groups were rapidly injected around the graft vessels, according to the instructions by manufacturer.

### Statistical analysis

All data were determined from multiple individual biological samples and presented as mean values ± standard error of the mean. The ''n'' represents the number of biological replicates indicated in the manuscript. All mice were randomly assigned to groups (male and female). The investigators analyzing the samples were blinded to the group allocations. Sample size estimates were not performed. For statistical comparisons, an unpaired two-sided Student's t-test was performed using GraphPad Prism software to compare differences between the two groups. An analysis of variance test was performed for over two groups. Statistical significance was considered at *p* < 0.05. All mice were randomly assigned to different experimental groups.

### Data availability

The accession number for the Single-cell RNA sequencing data of allografted arteries reported in this article is GSE211731. The bulk-RNA sequencing data and the single-cell RNA sequencing data of sorted tdTomato-positive cells are available in Gene Expression Omnibus (GSE will be accessible after formal publication). Any additional information required for the reanalysis of the data reported in this paper can be obtained from the lead contact upon request.

## Results

### Heterogeneity of CD34^+^ cells in transplant arteriosclerosis

**Figure 1:**
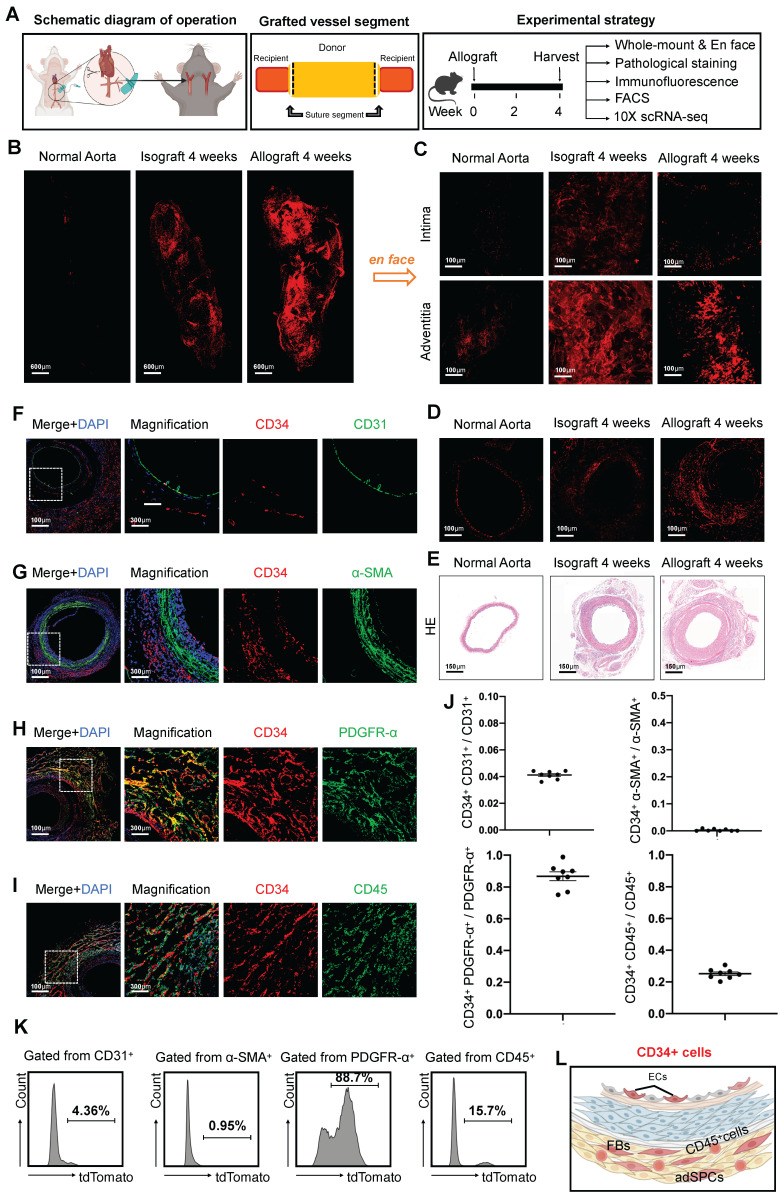
** Characterization of CD34^+^ cells in transplant arteriosclerosis.** (A) The schematic diagram of arterial graft model (left); Zone map for grafted artery segment (middle); An overview of the *in vivo* experiment strategy (right). (B) The whole-mount immunostaining of CD34 in normal thoracic aortas (n = 6), isografts (n = 6) and allografts (n = 6). (C) The en-face immunostaining of CD34 in normal thoracic aortas (n = 6), isografts (n = 6) and allografts (n = 6). (D)Representative immunostaining images of CD34 on cryosections from normal thoracic aortas (n = 6), isografts (n = 6) and allografts (n = 6). (E) Representative HE staining images from normal thoracic aortas (n = 6), isografts (n = 6) and allografts (n = 6). (F-I) Representative co-immunofluorescence images of CD34 and the endothelial cell marker CD31, smooth muscle cell marker α-SMA (α-smooth muscle actin), fibrotic cell marker PDGFR-α (platelet-derived growth factor receptor α), and inflammatory cell marker CD45 in 4-week grafted arteries (n = 8). Nuclei were counterstained with DAPI. (J) Semi-quantification of the percentage of CD34^+^ cells that express CD31, α-SMA, PDGFR-α and CD45. Data were presented as the mean ± SEM. (K) Flow cytometry analysis of the proportion of CD31, α-SMA, PDGFR-α and CD45 positive cells expressing CD34 in 4-week grafted arteries (n = 6). (L) Cartoon image showing that CD34^+^ cells include a subset of endothelial cells (ECs), adventitial stem/progenitor cells (adSPCs), CD45^+^ cells and fibroblasts (FBs).

To investigate changes in CD34 expression and its distribution patterns in the context of organ transplantation, we developed allograft models using cervical tail-cuff technology and established a comprehensive experimental strategy (Figure 1A). Since vascular remodeling generally stabilizes around four weeks post-transplantation 5, we evaluated CD34 expression in transplanted arteries at this time point. This was compared to normal thoracic aortas and isografts from three distinct perspectives: whole-mount view, intimal and adventitial surfaces, and cross-sectional analysis. Notably, four weeks after transplantation, we observed a marked upsurge in CD34 expression throughout the blood vessel (Figure 1B). To clarify the distribution of CD34 protein, we performed en-face staining on the endothelial and adventitial surfaces of the vessels. The staining results revealed a more pronounced increase in CD34 expression on the adventitial surface (Figure 1C). Moreover, immunohistochemical staining of sections indicated a more significant enlargement of CD34 on the adventitial surface compared to the intimal layer (Figure 1D). Consistent with previous studies 28, pathological hematoxylin and eosin (H&E) staining indicated neointima formation and adventitial hyperplasia in the remodeled vessels (Figure 1E). To further investigate the heterogeneity of CD34^+^ cells, we next conducted immunostaining on the transplanted arteries (Figures 1F-I). Furthermore, immunofluorescence staining revealed that the co-staining proportion of CD34 with the endothelial marker CD31 was only 4.1% (Figures 1F and J). There was minimal co-staining with the smooth muscle marker α-SMA (α-smooth muscle actin) (Figures 1G and J). The co-staining proportion with the fibroblast marker PDGFRα (platelet-derived growth factor receptor α) was as high as 86.8% (Figures 1H and J), while CD45^+^ lymphocytes were about 25.2% (Figures 1I and J). Subsequent digestion of the arteries four weeks after transplantation followed by flow cytometric analysis revealed that the percentages of CD31^+^, α-SMA^+^, PDGFRα^+^, and CD45^+^ cells among CD34^+^ cells were 4.36%, 0.95%, 88.7%, and 15.7%, respectively (Figure 1K). We found that CD34 primarily constituted adventitial stem or progenitor cells, fibroblasts, CD45^+^ inflammatory cells, and a small number of ECs in the intima and negligible expression in SMCs (Figure 1L).

We have previously systematically described inflammatory cells in allograft and isograft 29. To uncover the intrinsic features and role of CD34 in noninflammatory cells within grafted arteries, we selected CD45^-^ noninflammatory cells from four-week grafts and merged them with the public normal murine thoracic aorta dataset (GSE211731). All cells were clustered into 11 populations and were identified based on their biological specific markers (Figures S1A and D). Compared with normal thoracic aorta, there was a significant increase in the proportions of fibroblasts and ECs in the aortic graft (Figures S1B and C). Intriguingly, in previous experiments on arterial injury in mice, CD34 was also primarily localized in fibroblasts and ECs 26. To further investigate the heterogeneity of CD34, we subdivided CD34^+^ cells, which are constituted of 6 clusters of fibroblasts and 3 clusters of ECs (Figure S1E). The fibroblast subgroup (Cluster 1) with the most significant CD34 expression, upon gene ontology (GO) analysis, was associated not only with traditional extracellular matrix organization and wound healing but also more extensively with chemotaxis and cytokine-associated function of regulating angiogenesis (Figures S1F and G). Volcano plot analysis compared Cluster 1 to other fibroblast clusters further indicated upregulated genes primarily involved in stem cell markers (*Cd34/Sca1/Pi16/Cd44*) and chemokines (*Ccl2/Ccl11/Cxcl1/Cxcl2*) (Figure S1H). The sequencing results suggested that the subgroup of CD34^high^ fibroblasts plays a role in regulating vascular remolding in graft vasculopathy. These results collectively indicated that, although CD34 was previously thought to be a specific hematopoietic stem cell marker, CD34^+^ cells in the vessel wall are a heterogeneous population.

### Arterial CD34^+^ cells stimulate neointimal hyperplasia of transplant arteriosclerosis

**Figure 2:**
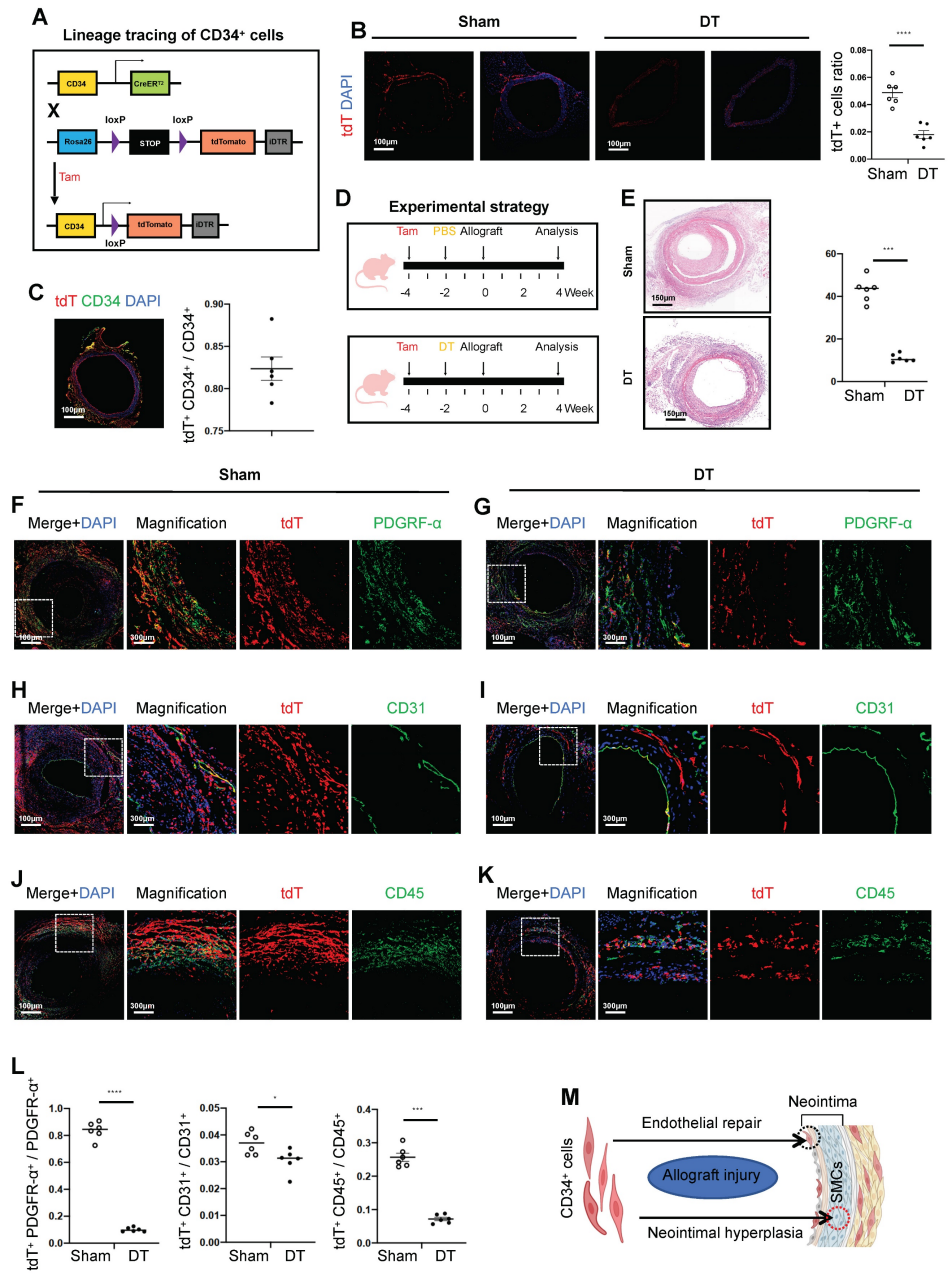
** Arterial CD34^+^ cells affect neointimal hyperplasia in the development of allograft arteriosclerosis.** (A) Schematic showing genetic lineage tracing by CD34-CreER^T2^; Rosa26-tdTomato; iDTR. (B) Representative immunofluorescence images of tdT^+^ cells from cryosections of CD34-CreER^T2^; Rosa26-tdTomato; iDTR mice treated with (n = 6) or without (n = 6) DT. (C) Representative co-immunofluorescence images of tdTomato and CD34 in CD34-CreER^T2^; Rosa26-tdTomato; iDTR mice (n = 6); and semi-quantification of the percentage of tdTomato^+^ cells in CD34^+^ cells. Data were presented as the mean ± SEM. (D) Sketch of the experimental strategy. (E) Representative HE staining of grafted arteries (n = 6); and semi-quantification of neointimal area in the presence and ablation of CD34^+^ cells. Data were presented as the mean ± SEM and analyzed by using an unpaired two-tailed Student's t-test. (F-K) Representative co-immunofluorescence images of CD34 and PDGFR-α, CD31, and CD45 on cryosections from 4-week grafted arteries in the group with (n = 6) and without (n = 6) DT. (L) Semi-quantification of the percentage of PDGFR-α, CD31 and CD45 cells expressing tdTomato for F-K. Data were presented as the mean ± SEM and analyzed by using an unpaired two-tailed Student's t-test. * p < 0.05, ** p < 0.01, *** p < 0.001, **** p < 0.0001. (M) Cartoon image showing that CD34^+^ cells repair endothelial cells and promote neointimal thickening in allograft injury.

To explore the role of CD34^+^ cells in arterial wall remodeling, we generated a CD34CreER^T2^ genetic lineage tracing mouse model. Crossing this model with Rosa26-tdTomato and diphtheria toxin receptor (DTR) gene mice, we obtained the CD34CreER^T2^; Rosa26-tdTomato-DTR mouse line (Figure 2A). In the group treated with diphtheria toxin (DT), CD34^+^ cells labeled with tdTomato-positive protein decreased by 63.3%, as confirmed by immunofluorescence staining (Figure 2B). Meanwhile, the labeling efficiency of tdtomato fluorescent protein reached 82.4% in the group without DT (Figure 2C). To assess the impact of CD34^+^ cell depletion on neointimal formation, we treated CD34CreER^T2^; Rosa26-tdTomato-DTR mice with tamoxifen and DT before arterial transplantation (Figure 2D). Histological analysis revealed a significant reduction in neointimal and adventitial areas in the DT-treated group, with a 74.4% decrease in neointimal area (Figure 2E). These findings demonstrated the critical role of CD34^+^ cells in neointimal formation following arterial transplantation.

In the transplantation cohort untreated with DT, the percentages of tdTomato and double-positive cells for PDGFRα, CD31, and CD45 were recorded at 83.7%, 3.7%, and 25.7%, respectively (Figures 2F, H, and J). Conversely, in the group treated with DT, these proportions significantly decreased to 10.1%, 3.0%, and 7.2%, respectively (Figures 2G, I, and K). The DT-treated group exhibited a significant reduction in fibroblasts and inflammatory cells originating from CD34, while changes in CD34-derived ECs were minimal (Figure 2L). In summary, the most pronounced decline in PDGFRα-positive fibroblasts and CD45-positive inflammatory cells derived from CD34 cells was observed following the genetic ablation of CD34 *in vivo*. This provides compelling evidence that CD34 lineage cells predominantly contribute to the population of fibroblasts and inflammatory cells, thereby significantly influencing neointimal hyperplasia (Figure 2M).

### Bone marrow CD34^+^ cells differentiate into inflammatory cells

In our subsequent investigation, we aimed to determine the origin of recipient CD34^+^ cells, specifically whether they are derived from bone marrow or non-bone marrow tissues. To assess the involvement of bone marrow-derived CD34 cells in the remodeling of the vascular wall during organ transplantation, we conducted transplantation of bone marrow from CD34CreER^T2^; Rosa26-tdTomato mice into normal C57BL/6J wild-type mice, with treatment intervals of two weeks (Figure S2A). Immunofluorescence analysis indicated minimal bone marrow-derived CD34 cells within the vascular wall. No significant change was observed in the thickness of the neointima (Figure S2B). The proportions of tdTomato and PDGFRα, CD31, and CD45 double-positive cells were recorded at 2.5%, 3.9%, and 18.8%, respectively (Figure S2C). Based on these findings, we concluded that a small fraction of bone marrow-derived CD34 cells may localize within the vascular wall, predominantly differentiating into CD45-positive inflammatory cells, with negligible impact on the neointima (Figure S2J).

To further explore the role of CD34^+^ cells within the arterial wall, we irradiated CD34CreER^T2^; Rosa26-tdTomato-DTR mice with X-rays to deplete their bone marrow cells and then transplanted them with bone marrow from normal C57BL/6J mice. After bone marrow reconstitution, we treated mice with tamoxifen and DT (or PBS as a control) before arterial transplantation (Figures S2D and F). In the control group, the proportions of tdTomato-positive cells co-stained with PDGFRα, CD31, and CD45 were 85.7%, 3.9%, and 22.3%, respectively. These proportions decreased to 10.2%, 3.3%, and 5.6% in the DT-treated group (Figures S2E, G, and H). Histological analysis revealed a 57.5% reduction in neointimal area in the DT-treated group (Figure S2I). Our findings suggested that a subset of CD34^+^ cells within the arterial wall functions as stem or progenitor cells, contributing to the replenishment of fibrotic cells. Besides, another subset of CD34^+^ cells may influence smooth muscle cell behavior (Figure S2J).

### ScRNA-sequencing analysis of CD34 lineage cells in the vessel wall

To investigate the fate of CD34^+^ cells, we generated CD34CreER^T2^; Rosa26-tdTomato transgenic mice and established vascular transplantation models. We isolated single-cell suspensions from vascular grafts four weeks after transplantation, sorted tdTomato-positive cells, and performed 10× sequencing. Unsupervised clustering analysis identified 10 cell clusters, including fibroblasts, ECs, inflammatory cells, and glial cells (Figures S3A and C). Given the predominance of fibroblasts within the CD34 lineage, we further re-clustered this population into seven subgroups (Figure S3B). We identified subclusters 1 and 3 expressing higher levels of *Cd34* and progenitor markers such as *Ly6a, Pi16,* and *Tek*. Conversely, other subclusters with lower *Cd34* expression and elevated levels of *Ccl11* and *Cxcl12* were characterized as chemotactic fibroblasts, while those expressing *Postn* and *Acta2* were identified as myofibroblasts. We reconstructed a fibroblast pseudotime trajectory to explore potential differentiation trajectories using DDRtree (Figure S3D). Cluster 0, with high *Cd34* expression, was positioned at the root of this trajectory, suggesting its role as a progenitor population.

To investigate the biological alterations along the differentiation trajectory, we screened 300 genes that exhibited the most significant changes along the pseudotime continuum. These genes were categorized into three distinct clusters based on their expression levels. Cluster 1, characterized by high expression at the initiation of the pseudotime axis, was notably enriched in biological processes associated with angiogenesis, fat and chondrocyte differentiation, and chemotaxis. Conversely, clusters 2 and 3 demonstrated elevated expression levels at the terminal end of the pseudotime axis, with their enriched processes primarily related to the extracellular matrix and collagen fibril formation (Figure S3E). We observed that chemotactic functions were particularly enriched among the early-stage genes. Therefore, we further investigated the chemotactic factors that were prominently expressed during the initial time points along the pseudotime axis (Figure S3F). Our analysis revealed that chemotactic factors displayed expression heterogeneity at various stages of fibroblast development, categorizing them into three main groups: the progenitor group (subclusters 1 and 3), the chemotactic group (subclusters 0 and 6), and the ECM-secretory group (subclusters 2, 4, and 5) (Figure S3G). In summary, bone marrow-derived CD34^+^ cells predominantly differentiated into CD45^+^ inflammatory cells. Moreover, a smaller subset of non-bone marrow-derived CD34^+^ cells can differentiate into ECs, while a larger proportion is primarily involved in chemotaxis related to early transplant immunity.

### PI16 expressing CD34 cells with different functions

**Figure 3:**
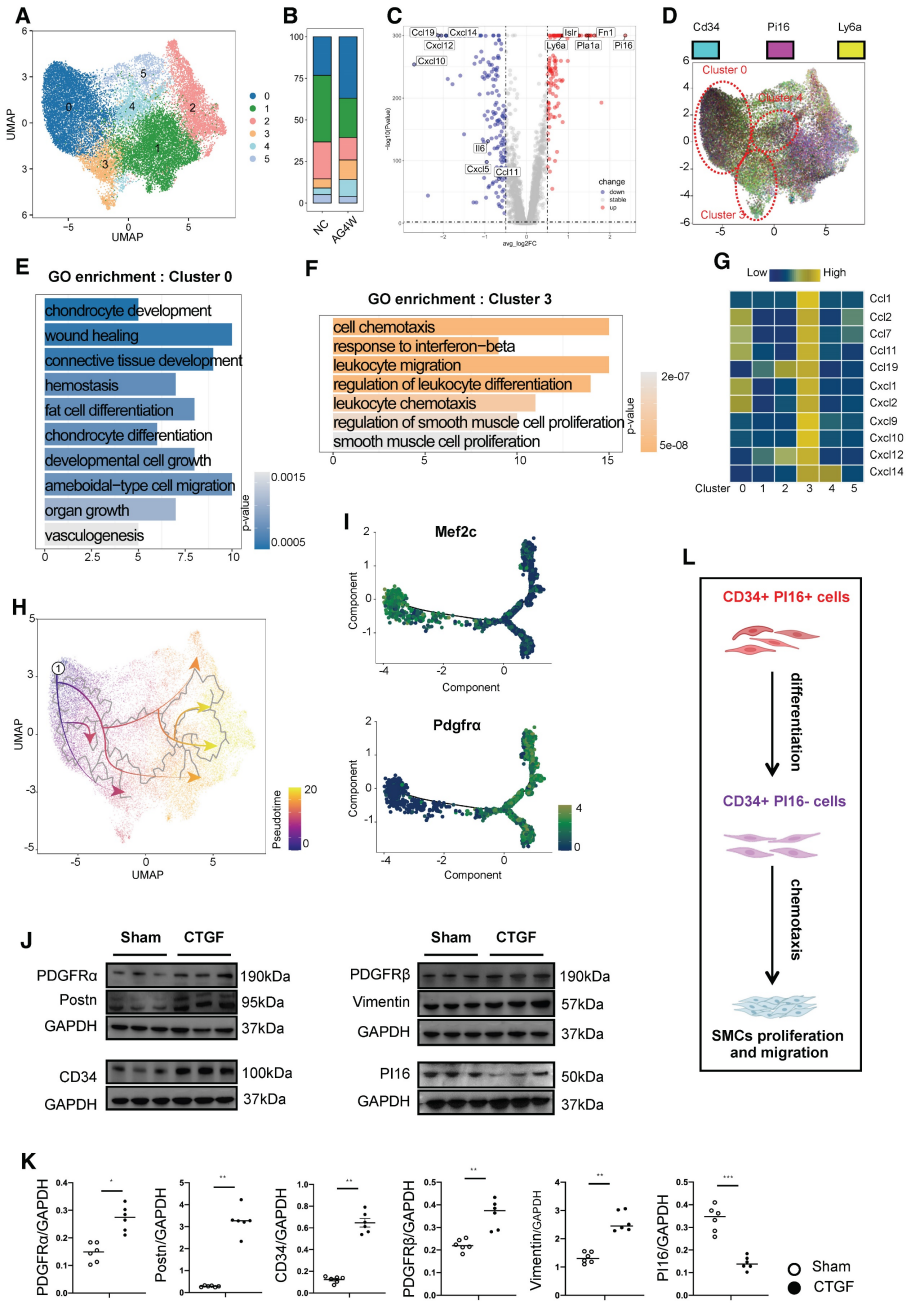
** CD34^+^ PI16^+^ SPC subpopulation and the pro-inflammatory effect of CD34^+^ PI16^-^ cell subpopulation.** (A) UMAP plots showing the major cell clusters of CD34^+^ cells in the normal thoracic aortas and grafted arteries (n = 43178 cells). (B) Bar chart showing the proportion of identified cell clusters in the normal thoracic aortas and grafted arteries. (C) Volcano plots showing the up-regulated gene for clusters 0 and 3. (D) Feature plots showing CD34 and PI16 expression in different clusters. (E) Gene Ontology analysis of top enriched functions in cluster 0. (F) Gene Ontology analysis of top enriched functions in cluster 3. (G) Analysis of the correlation between each cluster and the chemokine family. (H) Pseudotime trajectories showing CD34^high^ and PI16^high^ cells differentiated into endothelial cells and fibroblasts in the normal thoracic aortas and grafted arteries. (I) Trajectory plot showing the pseudotime representing trajectories of Mef2c^+^ and PDGFR-α^+^ cells differentiation and the expression of 2 markers (CD34/Ly6a) in the endothelial cells. (J) Representative western blot images of these markers (PDGFR-α/Postn/CD34/PDGFR-β/Vimentin/PI16) from CD34^+^ PI16^+^ cells treated with PBS and CTGF 50ug/L for 48 h. n = 6 per group. (K) Quantification analysis of these markers in (J). Data were presented as the mean ± SEM and analyzed by using an unpaired two-tailed Student's t-test. * p < 0.05, ** p < 0.01, *** p < 0.001, **** p < 0.0001. (L) Cartoon showing that CD34^+^ PI16^-^ cells, derived from CD34^+^ PI16^+^ cells, have a chemotactic effect on SMCs.

In our single-cell database of vascular grafts, we classified cells expressing *Cd34* into six distinct subgroups (Figure 3A). Furthermore, cells were stratified based on high and low expression levels, determined by the mean expression of *Cd34*. An analysis of the proportion of cells exhibiting elevated *Cd34* expression within each cluster revealed that clusters 0, 3, and 4 exhibited a higher percentage of *Cd34*-high expressing cells, with cluster 0 demonstrating an almost complete prevalence of *Cd34*-high expressing cells (Figures S4A and B). In comparison to clusters 0 and 3, genes such as *Fn1, Pi16, Ly6a, Pla1a,* and *Islr* were significantly upregulated in the high *Cd34* expression group, whereas genes including *Ccl11, Cxcl5, Il6, Ccl19, Cxcl12, Cxcl10,* and *Cxcl14* were significantly downregulated (Figure 3C). The *Pi16*-positive cluster, recognized as a crucial fibroblast progenitor, can differentiate into various specialized fibroblast types under pathological conditions, facilitating repair functions. Our database indicated that cells within clusters 0, 3, and 4 exhibiting high *Cd34* expression exhibited a notable increase in their proportion following vascular transplantation (Figure 3B), with cluster 0 also displaying elevated *Pi16* expression, while *Pi16* expression in cluster 3 was significantly diminished (Figure 3D). GO functional enrichment analysis indicated that cells in cluster 0 were primarily associated with processes such as chondrocyte development and differentiation, wound healing, connective tissue development, adipocyte differentiation, and angiogenesis (Figure 3E). Conversely, cluster 3 cells were predominantly linked to cell chemotaxis, the interferon-beta response, the regulation of lymphocyte migration and differentiation, and the proliferation and regulation of SMCs (Figure 3F). Moreover, cluster 4 cells were associated with wound healing, the regulation of cell-matrix adhesion, and collagen fiber formation (Figure S4D). Furthermore, cluster 3 cells exhibited the strongest correlation with genes related to chemotactic factors, including *Ccl1, Ccl2, Ccl7, Ccl11, Ccl19, Cxcl1, Cxcl2, Cxcl9, Cxcl10, Cxcl12,* and *Cxcl14* (Figure 3G). The observed variations in wound healing results among mice of different ages can be attributed to the secretory activity of inflammatory fibroblasts. A similar study also found that variations in wound healing outcomes across mice of different ages are attributable to the secretory activity of inflammatory fibroblasts 30.

Consistent with previous findings, cluster 0 demonstrated a differentiation potential into other cell types (Figure 3H). Based on the observed angiogenic potential of cluster 0 cells, we performed DDRtree dimensionality reduction jointly on this cluster and the EC cluster (Figure S4C). Two distinct branches emerged in the differentiation trajectory, with *Mef2c* expression higher in state 1 and *Pdgfra* expression higher in states 2-7 (Figure 3I). Vascular progenitor cell markers (*Cd34* and *Ly6a*) were predominantly expressed in group 0 fibroblasts and gradually decreased during differentiation into ECs (Figure S4E). We hypothesized that cluster 0 cells with high expression of *Cd34* and *Pi16* represent a progenitor cell subgroup, while cluster 3 is a chemotactic fibroblast subpopulation. For verification, we generated PI16CreER^T2^ transgenic mice, administered tamoxifen, and isolated primary adventitial cells from the thoracic aorta. After sorting tdTomato-positive and CD34-positive cells, we stimulated them *in vitro* with connective tissue growth factor (CTGF). Protein electrophoresis experiments revealed that fibroblast-related proteins (*Pdgfra/Pdgfrb/Vimentin/Postn*) were significantly upregulated compared to the control group, while CD34 protein increased and PI16 protein decreased under CTGF stimulation. These results support the notion that CD34 and PI16 double-positive cells can differentiate into CD34-positive and PI16-negative cells, representing a stem or progenitor cell population (cluster 0) that can form a chemotactic fibroblast subpopulation (Figures 3J and K). The inflammatory chemotactic function of cluster 3 may be crucial in promoting smooth muscle cell proliferation and migration (Figure 3L).

### PI16^+^ cell fate in transplant arteriosclerosis

To investigate the lineage of PI16-positive cells further, we generated PI16CreER^T2^ lineage tracing mice and used tamoxifen by intragastric administration (Figure S5A). Immunofluorescence analysis of aortic frozen sections revealed that tdTomato-positive areas co-stained with PI16 protein accounted for only 4.7% in the normal aorta group but increased to 58.6% in the transplantation group (Figure S5B). Some PI16 lineage cells overlapped with CD34-positive cells (Figure S5C). Before injury, the expression of CD34 and PI16 in the blood vessel wall was low (Figure S5B). However, four weeks after surgery, PI16 lineage cells within the blood vessel wall increased significantly, including some CD34^+^ cells, with an overlap of 12.4% (Figure S5D). Our single-cell analysis revealed that PI16 expression was predominantly localized to fibroblasts (Figures S5E and F), with associated functions including angiogenesis, cell-matrix adhesion, wound healing regulation, and adipocyte differentiation (Figure S5G). In the vascular transplant group, we stained for EC markers (CD31), smooth muscle cell markers (a-SMA), fibroblast markers (PDGFRα), and inflammatory cell markers (CD45) to assess their colocalization with tdTomato. The results indicated that PI16 lineage cells primarily resided in adventitial fibroblasts and participated in vascular remodeling in transplant vasculopathy, with colocalization rates of 11.5%, 3.2%, 75.1%, and 1.2% with CD31, a-SMA, PDGFRα, and CD45, respectively (Figures S5H-K).

### CD34^+^ PI16^+^ cells serve as fibroblast progenitor cells

**Figure 4:**
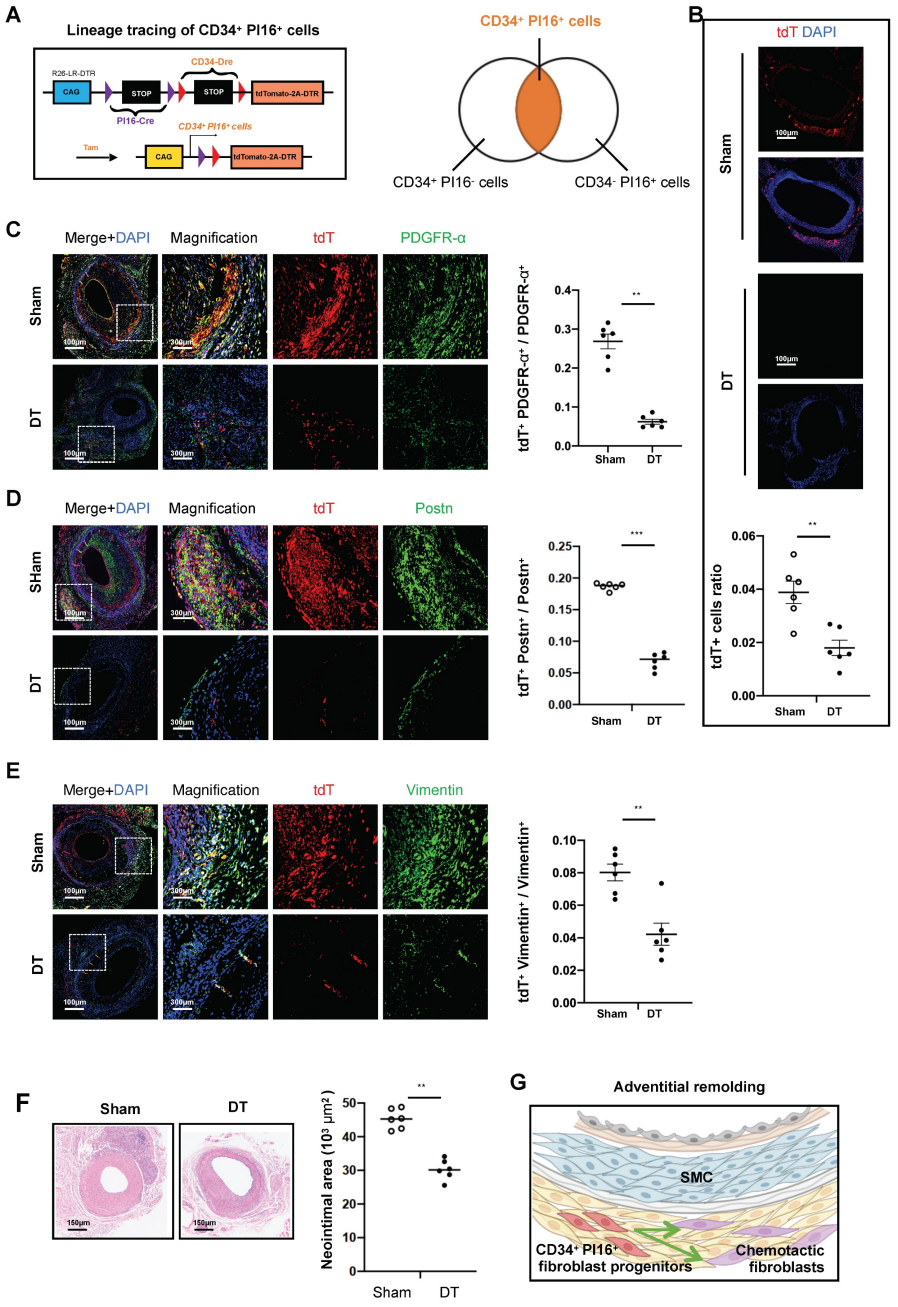
** CD34^+^ PI16^+^ stem/progenitor cells participate in fibroblastic repair.** (A) Schematic showing dual-recombinase-activated lineage tracing by CD34-Dre and PI16-CreER^T2^. (B) Representative immunofluorescence images of tdT^+^ cells from cryosections of CD34-Dre; PI16-CreER^T2^; Rosa26-tdTomato-iDTR mice treated with (n = 6) or without (n = 6) DT. (C-E) Representative co-immunofluorescence images of tdTomato and PDGFR-α, Postn, and Vimentin on cryosections from 4-week grafted arteries in the group with (n = 6) and without (n = 6) DT. Right panel: semi-quantification of the percentage of PDGFR-α, Postn and Vimentin cells expressing tdTomato for C-E. Data were presented as the mean ± SEM and analyzed by using an unpaired two-tailed Student's t-test. (F) Representative HE staining of grafted arteries from CD34-Dre; PI16-CreER^T2^; Rosa26-tdTomato-iDTR mice treated with (n = 6) or without (n = 6) DT. Right panel: semi-quantification of neointimal area in the presence and ablation of CD34^+^ and PI16^+^ dual-positive cells. Data were presented as the mean ± SEM and analyzed by using an unpaired two-tailed Student's t-test. * p < 0.05, ** p < 0.01, *** p < 0.001, **** p < 0.0001. (G) Cartoon image showing that CD34^+^ PI16^+^ SPCs participate in adventitial remolding.

Based on preliminary animal studies and single-cell analyses, identifying a specific subgroup within CD34 cells exhibiting enhanced progenitor cell characteristics has emerged as a critical focus. To facilitate the transcriptional categorization of CD34 cells into progenitor and other specialized subgroups, we employed the PI16 molecule. This approach led to the development of CD34-Dre and PI16-Cre dual recombinase lineage tracing mice, which were subsequently bred to produce mice harboring the Rosa26-tdTomato-DTR reporter gene (Figure 4A). Following tamoxifen induction, the mice were allocated into two experimental groups, including one receiving saline and the other administered DT. In the group treated with DT, CD34, and PI16, dual positive cells labeled with tdTomato-positive protein decreased by 53.8%, compared with the group treated with PBS (Figure 4B). To elucidate the role of these cells in adventitial reconstruction, we conducted validation using three fibroblast-specific antibodies, including Pdgfra, Postn, and Vimentin. Four weeks after modeling, the proportion of tdTomato and Pdgfra double-positive cells constituted 26.8% of PDGFRα-positive cells in the non-DT group, which diminished to 6.2% following DT treatment (Figure 4C). Similarly, tdTomato and Periostin double-positive cells in the non-DT group represented 18.7% of Postn-positive cells, decreasing to 6.9% post-DT treatment (Figure 4D). Furthermore, tdTomato and Vimentin double-positive cells accounted for 8% of Vimentin-positive cells in the non-DT group, which reduced to 4.2% after DT treatment (Figure 4E). Besides, H&E staining revealed that the neointimal area measured 45.3*10^3^ µm^2^ in the non-DT group, which decreased to 30.2*10^3^ µm^2^ following DT treatment, reflecting a reduction of 33.3% (Figure 4F). Conversely, a 74.4% reduction in neointimal area was observed in experiments where CD34 cells were eliminated (Figure 2E). We propose that the CD34 cell subgroup characterized by low PI16 expression exerts a chemotactic influence on SMCs, while the subgroup with high PI16 expression functions as a progenitor cell population involved in the repair of adventitial fibroblast damage within the vascular adventitia (Figure 4G).

Based on the above experimental findings, we suggest that CD34^+^ PI16^+^ cells can serve as a storage pool for adventitial fibroblasts and reconstruct the adventitia through differentiation and self-expansion. Gross staining of the transplanted vessels at 1, 2, 3, and 4 weeks revealed that the expression of CD34 and Pdgfra gradually increased (Figure S6A). At four weeks after transplantation, single-cell data demonstrated that the various fibroblast groups in the vessel were closely related to ECM deposition and calcification (Figure S6B). Moreover, H&E staining revealed that the thickness of the adventitia and neointima gradually increased from weeks 1 to 4. Masson and Sirius Red staining indicated that the collagen deposition in the adventitia and neointima also gradually increased over weeks 1 to 4 (Figure S6C).

### The role of CD34^+^ PI16^-^ chemotactic fibroblast subset

**Figure 5:**
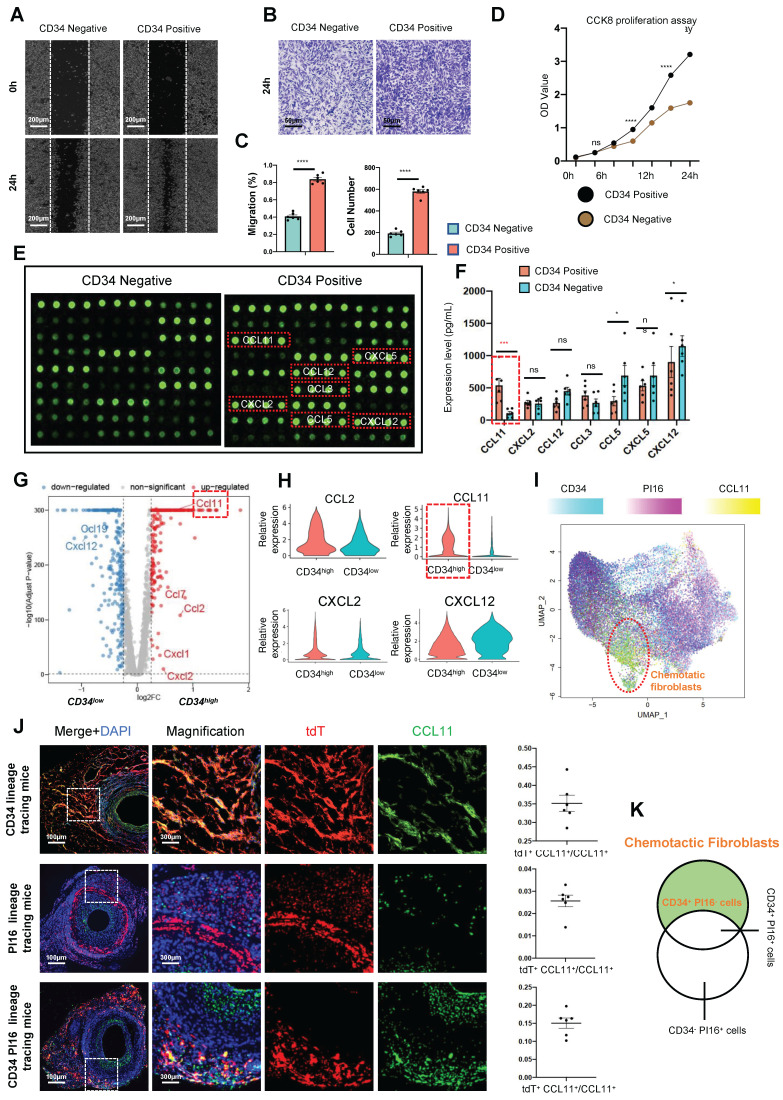
** Inflammatory chemotaxis of CD34^+^ and PI16^-^ cell subpopulations on smooth muscle cells.** (A) Representative scratch images of SMCs cultured with CD34 negative cell supernatants and CD34 positive cell supernatants for 24 hours. n = 6 per group. (B) Representative images of migrating SMCs cultured with CD34 negative cell supernatants and CD34 positive cell supernatants for 24 hours. n = 6 per group. (C) Quantification analysis of scratch-healing and migration rates of SMCs between CD34^-^ cell supernatants group and CD34^+^ cell supernatants group. (D) CCK8 proliferation assay of SMCs cultured with CD34 negative cell supernatants group and CD34 positive cell supernatants. n = 6 per group. (E) Protein microarray of chemokine showing that the difference among CD34^+^ cell supernatants group and CD34^-^ cell supernatants group. n = 6 per group. (F) Elisa experiments showing the expression level of selecting chemokines among CD34^+^ cell supernatants group and CD34^-^ cell supernatants group. n = 6 per group. Data were presented as the mean ± SEM and analyzed by using an unpaired two-tailed Student's t-test. * p < 0.05, ** p < 0.01, *** p < 0.001, **** p < 0.0001. (G) Volcano plots showing the up-regulated gene of cell chemokines between CD34-high expressing cells and CD34-low expressing cells. (H) Violin plots showing the expression difference of 4 chemokines (CCL2/CCL11/CXCL2/CXCL12) between CD34-high expressing cells and CD34-low expressing cells. (I) Feature plots showing the distribution of CD34, PI16 and CCL11 genes. (J) Representative co-immunofluorescence images of tdTomato and CCL11 in three kinds of lineage tracing mice. n = 6 per group. And semi-quantification of the percentage of tdTomato^+^ cells in CCL11^+^ cells. Data were presented as the mean ± SEM. (K) Schematic showing the chemotactic role of CD34^+^ cells and PI16^-^ cells.

Functionally distinct fibroblast subgroups play a significant role in heart disease 15, 31. In our single-cell database, the relationship of cluster 3 (Figure 3A), which exhibits high *Cd34* and low *Pi16* expression, with cell chemotaxis remains unclear. To investigate the influence of the chemotactic fibroblast subgroup on SMCs, we aimed to stratify primary fibroblasts from the adventitia of transplanted vessels into CD34-positive and negative fractions using magnetic column separation. We centrifuged at 2000 rpm for 5 min to remove cellular debris and contaminants, then collected the supernatants from both cell populations. Post-scratch assay on smooth muscle cell lines, they were incubated for 24 h with supernatants from CD34-positive and -negative fibroblast cultures to assess the scratch-healing race of cells. We found that smooth muscle healing with CD34 positive cell supernatant was rapid, with a migration rate of 83.9%. However, the migration rate of smooth muscle cultured with CD34 negative cell supernatant was only 40.8% (Figures 5A and C). Furthermore, we established a pair of co-culture models featuring SMCs seeded in the upper chamber and CD34-positive or -negative fibroblasts positioned in the lower compartment. Following a 24-h co-culture period, SMCs on the reverse side of the translucent polycarbonate membrane were stained with crystal violet for microscopic examination and imaging. The average migration number of SMCs in the CD34 positive cell co-culture group was 579 cells compared to 193 cells in the Cd34 negative cell co-culture group (Figures 5B and C). The effect of CD34 positive and negative cells on the proliferation of SMCs was assessed using a CCK8 proliferation assay kit, revealing a steeper proliferation curve for the smooth muscle group treated with CD34^+^ cells (Figure 5D). The CD34^+^ cell niche exhibits significant plasticity, a portion of which is implicated in mediating inflammatory chemotaxis.

As described above, CD34^+^ cells from transplanted vessels significantly promote the proliferation and migration of vascular SMCs. We aimed to use a mouse chemokine microarray protein chip to clarify the differences in chemokine factors secreted by CD34 positive and negative cells. Proteomics results of chemokine factors revealed that seven chemokines were upregulated in CD34-positive cells cultured *in vitro* (*Ccl3/Ccl5/Ccl11/Ccl12/Cxcl2/Cxcl5/Cxcl12*), with *Ccl3/Ccl11/Cxcl5/Cxcl12* expression exceeded by more than 10-fold (Figure 5E). Meanwhile, enzyme-linked immunosorbent assay results of supernatants from cultured adventitial cells of transplanted vessels suggested that *Ccl3/Ccl11/Cxcl2* expressions were more significant in the CD34 positive group than the CD34 negative group (Figure 5F). Single-cell data *in vivo* also indicated that *Ccl11* expression was most significant in the *Cd34* high expression group within fibroblasts (Figures 5G and H). The *Ccl11* expression was predominantly concentrated in cluster 3 cells (Figure 5I), corresponding to the previously identified secretory subgroup (*Cd34* high expression, *Pi16* low group). To illustrate the relationship between CCL11 and this secretory subgroup, we used three lineage-tracing mouse models—CD34CreER^T2^-tdTomato, PI16CreER^T2^-tdTomato, and CD34Dre-PI16Cre-tdtomato-DTR—for the vascular transplant model. Four weeks post-transplantation, paraffin-embedded tissue sections were prepared and stained to visualize the expression and location of tdTomato and CCL11. Fluorescence microscopy revealed that the colocalization frequencies of CCL11 with CD34 lineage cells and CD34 PI16 lineage cells were 35.2% and 15%, respectively, while colocalization with PI16 lineage cells was infrequent (Figure 5J). The CD34 cell population, excluding PI16, is a key chemotactic subpopulation of fibroblasts (Figure 5K) and is capable of secreting various inflammatory factors, with CCL11 being specifically secreted by this population.

### Effects of CCL11-CCR3 on neointimal hyperplasia

In our vascular transplant model, we observed that the proliferation of SMCs coincided with the proliferation of adventitial components during the first to fourth weeks, occurring almost simultaneously (Figure S7A). A single-cell analysis of SMCs identified six distinct subgroups categorized into four primary biological classifications: chemotactic, secretory, and proliferative clusters. Notably, the proportion of secretory and chemotactic cells within the transplanted vessels significantly increased following transplantation (Figure S7B).

The heatmap illustrated the differential gene expression among the six subgroups, with Group 0 (contractile) demonstrating elevated expression levels of *Acta2, Igtb8, Cnn1,* and *Myh11*; Group 1 (chemotactic) exhibiting heightened expression of *Ccl1, Ccl11,* and *Meg3*; Groups 3 and 5 (secretory) expressing genes such as *Col1a, Col8a, Spp1,* and *Mmp3*; and Groups 2 and 4 (proliferative) expressing Fos, *Rbp4, Sparcl1, and Des,* among others (Figure S7C). Besides, gene ontology analysis of biological processes further delineated the characteristics of SMCs (Figure S7D). To investigate the type of SMCs that regenerate following arterial grafting, we established a pseudotime trajectory. Our findings indicated that cells in a normal state predominantly resided at branch 1, whereas cells post-grafting were primarily located at branches 2 and 3. This observation suggested that SMCs undergo two principal fate transitions after arterial grafting. One involved the production and response to chemotactic factors (branch 2), and the other related to collagen fibril secretion and participation in wound healing (Figures S7E and F).

**Figure 6:**
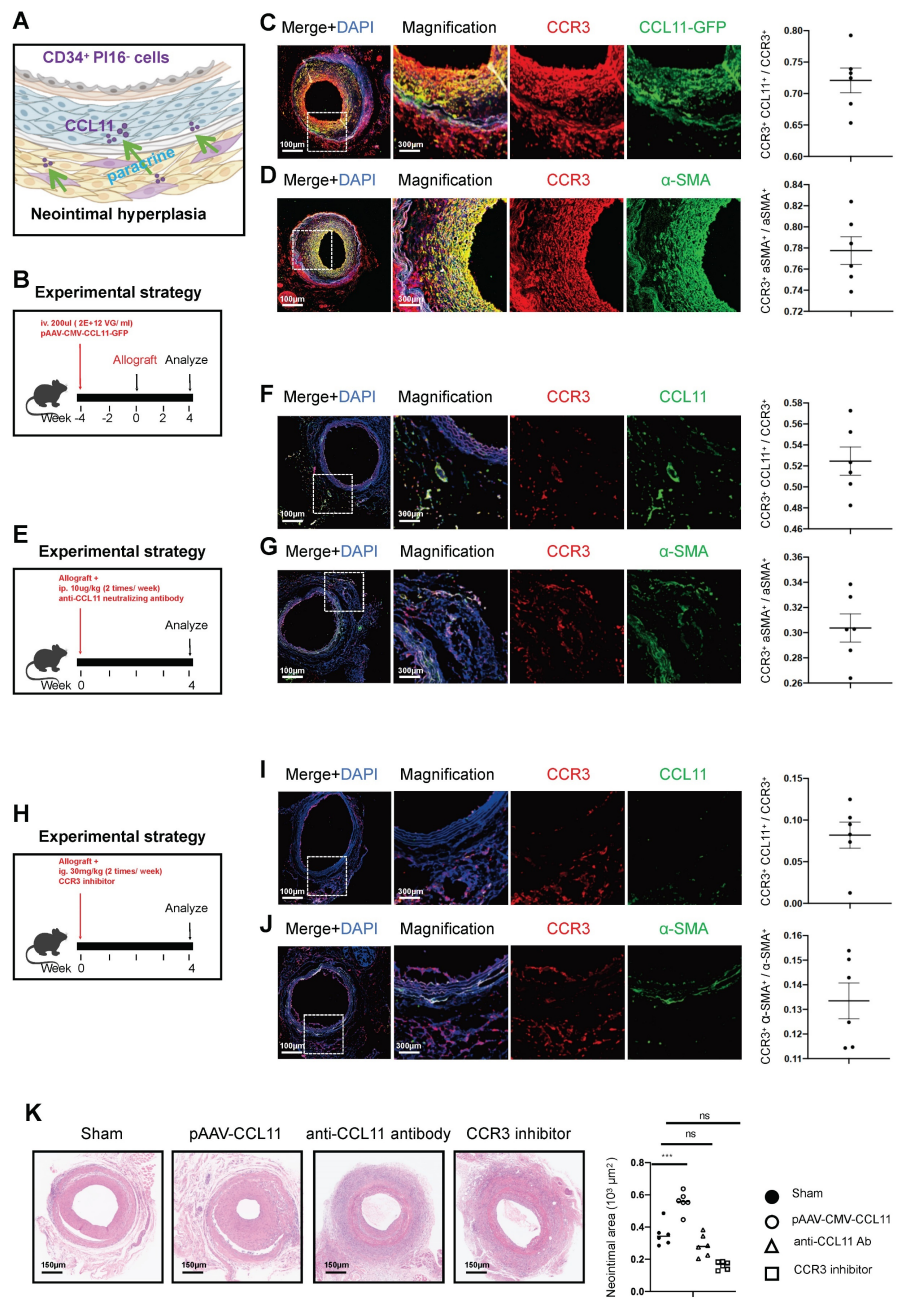
** Effects of chemokine CCL11 and its receptor CCR3 on neointimal hyperplasia in the aortic allografts.** (A) Cartoon image showing that CCL11 secreted by CD34^+^ PI16^-^ cells binds to the CCR3 on SMCs. (B/E/H) Sketch of the experimental strategy. (C) Representative co-immunofluorescence images of CCR3 and CCL11-GFP in grafted arteries with AAV. CCL11 infection (n = 6). And semi-quantification of the percentage of CCL11^+^ cells in CCR3^+^ cells. Data were presented as the mean ± SEM. (D) Representative co-immunofluorescence images of CCR3 and a-SMA in grafted arteries with AAV. CCL11 infection (n = 6). And semi-quantification of the percentage of CCR3^+^ cells in a-SMA^+^ cells. Data were presented as the mean ± SEM. (F) Representative co-immunofluorescence images of CCR3 and CCL11-GFP in grafted arteries with anti-CCL11 neutralizing antibody treatment (n = 6). And semi-quantification of the percentage of CCL11^+^ cells in CCR3^+^ cells. Data were presented as the mean ± SEM. (G) Representative co-immunofluorescence images of CCR3 and a-SMA in grafted arteries with anti-CCL11 neutralizing antibody treatment (n = 6). And semi-quantification of the percentage of CCR3^+^ cells in a-SMA^+^ cells. Data were presented as the mean ± SEM. (I) Representative co-immunofluorescence images of CCR3 and CCL11-GFP in grafted arteries with CCR3 inhibitor treatment (n = 6). And semi-quantification of the percentage of CCL11^+^ cells in CCR3^+^ cells. Data were presented as the mean ± SEM. (J) Representative co-immunofluorescence images of CCR3 and a-SMA in grafted arteries with CCR3 inhibitor treatment (n = 6). And semi-quantification of the percentage of CCR3^+^ cells in a-SMA^+^ cells. Data were presented as the mean ± SEM. * p < 0.05, ** p < 0.01, *** p < 0.001, **** p < 0.0001. (K) Representative HE staining of 4-week grafted arteries from the control group, AAV.CCL11 infection group, anti-CCL11 neutralizing antibody treating group and CCR3 inhibitor-treating group. n = 6 per group. Right panel: semi-quantification of neointimal area in these four groups. Data were presented as the mean ± SEM and analyzed by using an unpaired two-tailed Student's t-test.

The paracrine chemotactic effect of the CD34^+^ PI16^-^ cell group, especially represented by CCL11, promotes the proliferation of smooth muscle cells (Figure 6A). Thus, we engineered an adeno-associated virus for the overexpression of CCL11 and administered a 200ul injection (with a viral titer of 2*10^12^ VG/ml) via the tail vein four weeks before arterial transplantation. Four weeks of section staining revealed co-expression of CCL11 and CCR3 within the smooth muscle layer, with CCL11 and CCR3 co-localizing in 72.1% of cases, and CCR3 and α-SMA co-localizing in 77.8% of the observed instances (Figures 6B, 6C, and 6D). Additionally, CCL11 neutralizing antibodies were administered twice a week at 100ug/kg intraperitoneally 32. Four weeks on, a notable decline in CCL11 and CCR3 levels was observed, with their co-localization dropping to 52.5%, and the CCR3 and α-SMA co-localization falling to 30.4%, leading to a substantial reduction in smooth muscle proliferation (Figures 6E, 6F, and 6G). Therefore, we used a CCR3 inhibitor (Selleck, S3612) twice a week after vascular transplantation, at a dose of 30mg/kg each time. The results showed the colocalization proportion of CCL11 and CCR3 was 8.2%, and Ccr3 and α-SMA was 13.3%, with lighter proliferation in the smooth muscle layer (Figures 6H, 6I, and 6J). Upon comparing the sham group, the pAAV2/9-CMV-CCL11 treatment group, the CCL11 neutralization group, and the CCR3 inhibitor group, the mean neointimal thicknesses were recorded at 35.4*10^3^ um^2^*,* 55.7*10^3^ um^2^, 28.6*10^3^ um^2^*,* and 16.1*10^3^ um^2^, respectively (Figure 6K).

### CCL11 activates the PI3K-AKT pathway in smooth muscle cells

**Figure 7:**
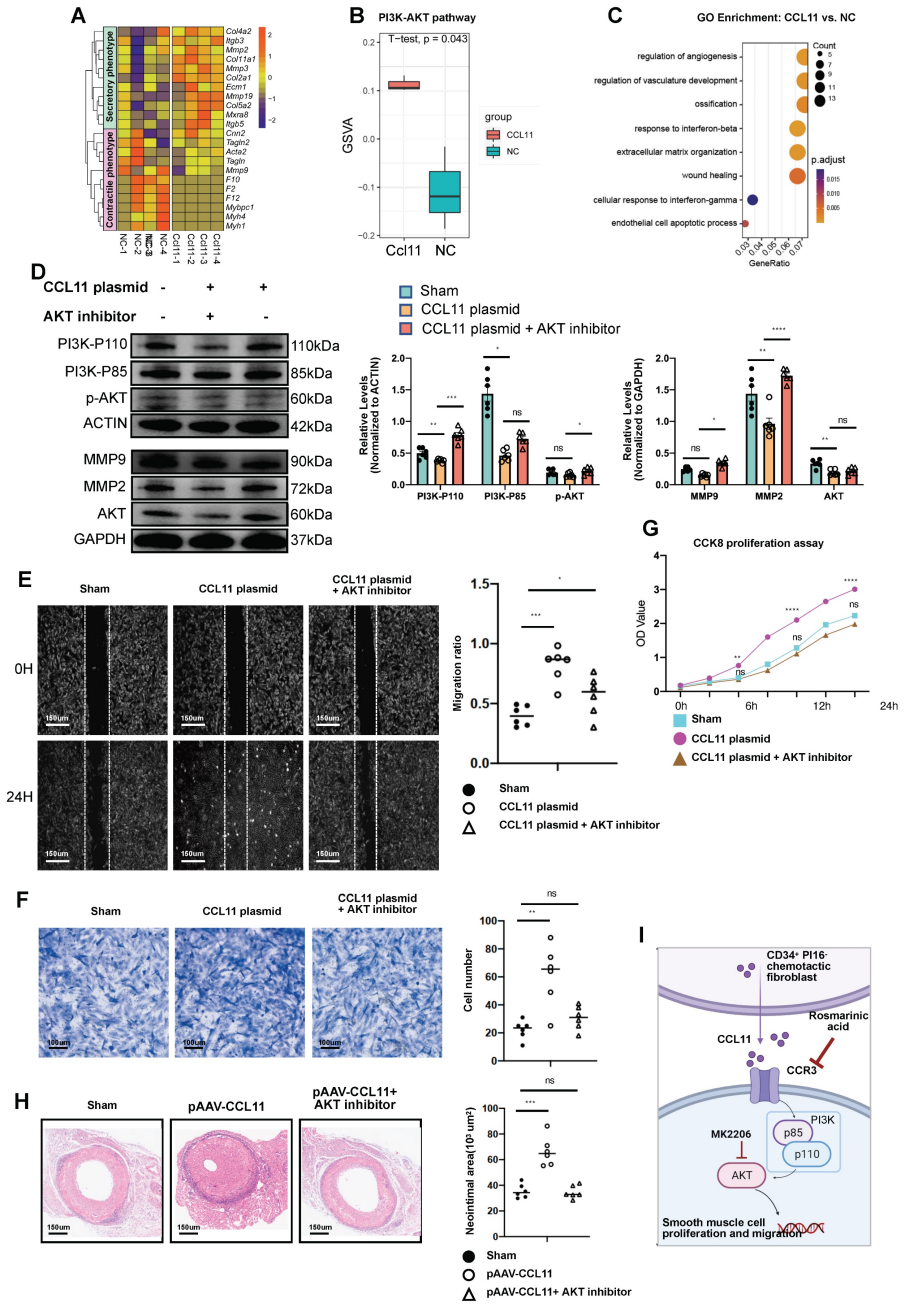
** CCL11 promotes smooth muscle proliferation and migration by activating the PI3K-AKT signaling pathway.** (A) Heatmap showing that contractile or secretory gene changes in SMCs from the control group and the CCL11 plasmid-stimulated group (n = 4 per group). (B) Violin plots showing that the up-regulated PI3K-AKT signal in the control group and the CCL11 plasmid-stimulated group (n = 4 per group). (C) GO enrichment showing that functional differences between smooth muscle cell lines and smooth muscle cells treated by CCL11. (D) Representative western blot images of these markers related to the PI3K-AKT pathway from SMCs treated with PBS, CCL11-plasmid and CCL11-plasmid plus AKT inhibitor for 12h (n = 6 per group). Right panel: quantification analysis of these markers. Data were presented as the mean ± SEM and analyzed by using an unpaired two-tailed Student's t-test. (E) Representative scratch images of SMCs cultured with PBS, CCL11-plasmid and CCL11-plasmid plus AKT inhibitor for 12h (n = 6 per group). Right panel: quantification analysis of scratch-healing rates of SMCs among these three groups. (F) Representative migration images of SMCs cultured with PBS, CCL11-plasmid and CCL11-plasmid plus AKT inhibitor for 12h (n = 6 per group). Right panel: quantification analysis of migration rates of SMCs among these three groups. (G) CCK8 proliferation assay of SMCs cultured with PBS, CCL11-plasmid and CCL11-plasmid plus AKT inhibitor for 12h (n = 6 per group). (H) Representative HE staining of 4-week grafted arteries from the control group, AAV.CCL11 infection group, and AAV.CCL11 infection plus CCR3 inhibitor-treating group. n = 6 per group. Right panel: semi-quantification of neointimal area in these four groups. Data were presented as the mean ± SEM and analyzed by using an unpaired two-tailed Student's t-test. * p < 0.05, ** p < 0.01, *** p < 0.001, **** p < 0.0001. (I) Cartoon image showing that binding of CCL11 to its receptor activates the PI3K-AKT signaling pathway.

In their unstimulated state, SMCs predominantly exhibit a contractile phenotype. However, stimulation with CCL11 induces a transition to a secretory phenotype, as evidenced by the upregulation of secretion-associated genes demonstrated in the heatmap analysis. (Figure 7A). In SMC lines transfected with CCL11-overexpressing plasmids, bulk-RNA sequencing revealed significant upregulation of the PI3K-AKT signaling pathway in the overexpression group (Figure 7B). GO functional enrichment analysis further indicated that the CCL11 overexpression group was closely associated with regulating processes, such as angiogenesis, ossification, Ifnb response, and ECM deposition (Figure 7C). In the immunoblotting experiment, we designed three groups, including an untreated group, a CCL11 overexpression group, and a CCL11 overexpression group with AKT inhibition. The results indicated an upregulation of P110 and P85 subunits, MMP2, MMP9, AKT, and p-AKT, in the CCL11 overexpression group. However, in the CCL11 overexpression and Akt inhibition group, the expression of these proteins was relatively decreased (Figure 7D). Subsequently, it is imperative to conduct an in-depth investigation into the mobility of smooth muscle. The smooth muscle samples were categorized into three distinct groups, including a control group, a group subjected to stimulation with the CCL11 plasmid, and a group that received stimulation with the CCL11 plasmid in conjunction with an AKT inhibitor (MK2206). To eliminate the influence of proliferation factors, mitomycin was incorporated into the smooth muscle culture medium before the commencement of the experiment (Figure 7E). Similar conclusions were drawn from the migration experiments (Figure 7F). The CCK8 cell proliferation assay results also indicated that cell proliferation was rapid with CCL11 overexpression, while AKT inhibition decreased proliferation (Figure 7G). Furthermore, *in vivo*, Fluronic F-127 (Sigma-Aldrich, P2443) gel was employed to individually coat an overexpression plasmid of CCL11 AAV and a composite of the same plasmid with an AKT inhibitor (Shelleck, MK2206). Subsequent H&E staining indicated neointimal thickness measurements of 35.5*10^3^ μm^2^, 66.4*10^3^ μm^2^, and 34.7*10^3^ μm^2^, consistent with the expected trend (Figure 7H).

Results from CCK8 cell proliferation assays suggested an expedited proliferation rate in cells with CCL11 overexpression, which was significantly reduced following CCR3 knockdown (Figure S8A). In the transwell migration assays, SMCs exhibited significantly enhanced healing rates after CCL11 overexpression. Conversely, this accelerated healing process was mitigated upon applying CCR3-siRNA (Figure S8B). This trend was consistently mirrored in the scratch-healing experiments (Figure S8C). Compared to normal fibroblasts, fibroblasts from transplanted vessels exhibited a significant increase in the RNA level of certain genes (SM22/α-SMA/PDGFRα/Vimentin/Postn) (Figure S8D). In SMC lines, after adding the CCL11 cytokine, the RNA levels of certain genes (*Sm22/αSma/ColI/Mmp2/Mmp9*) increased (Figure S8E). However, after adding the CCL11 cytokine and treatment with CCR3-siRNA, the RNA levels of specific genes (*Sm22/Mmp2/Mmp9*) significantly decreased (Figure S8F). Furthermore, we divided SMCs into three groups, including a control group, a CCL11 cytokine-treated group, and a CCL11 cytokine and CCR3-siRNA-treated group. Western blot representative conditions and semi-quantitative analysis indicated that the expression levels of CCL11/α-SMA proteins were upregulated in SMCs stimulated with CCL11 cytokine, while these protein expressions decreased in the group treated with CCR3-siRNA simultaneously (Figure S8G). Accordingly, CCL11 secreted by CD34^+^ PI16^-^ cells, upon binding with receptors such as CCR3, activates the PI3K-AKT signaling pathway in SMCs, leading to smooth muscle cell proliferation and migration (Figure 7I).

## Discussion

In this study, we used single-cell RNA sequencing, antibody microarray, and genetic lineage tracing to uncover the direct or indirect involvement of CD34^+^ progenitor cells in the remodeling and repair of allografts. We found that CD34^+^ cells in the vessel wall are a heterogeneous population that consists of ECs, CD45^+^ inflammatory cells, and fibrotic cells. Based on bone marrow transplantation and DT genetic ablation experiments, we found that the CD34^high^ PI16^high^ cell population does not become CD45^+^ inflammatory cells; however, it promotes adventitial remodeling after vessel transplantation. Moreover, CD34^high^ PI16^high^ can differentiate into CD34^high^ PI16^low^ population serving as cytokine-releasing cells, which is crucial in boosting smooth muscle cell expansion. Our findings indicated that CD34^high^ PI16^high^ cells are fibroblast progenitors responsible for not only adventitial fibrosis via differentiating into myofibroblasts but also cytokine-producing CD34^high^ PI16^low^ cells. Importantly, blocking the CCL11 cytokine-receptor binding resulted in a reduction of neointimal lesions, offering a new perspective for therapies for vascular disease in the future.

CD34^+^ cells have been utilized to enhance heart regeneration following myocardial infarction, although the treatment outcomes have been variable 33-35. Additionally, an anti-CD34 antibody-coated stent was used to promote endothelial repair after angioplasty. However, the long-term clinic trials did not support its beneficial effectiveness 36. A better understanding of the detailed mechanisms underlying the composition and functions of CD34^+^ cells is necessary. Recent studies indicated that bone marrow-derived CD34^+^ cells contributed to inflammatory cell formation, while small, non-bone marrow Cd34^+^ cells can differentiate into ECs 37-39. Our study found that CD34^+^ PI16^+^ cells in the tunica adventitia may act as fibroblast progenitors to participate in vascular fibrosis and differentiate into cytokine-producing cells to enhance neointima lesions. These findings of highly heterogeneous CD34^+^ cells, including endothelial regenerative and inflammatory cells and fibroblasts, indicate that a refinement of CD34^+^ cell-based therapy is imperative in clinical application.

Organ fibrosis is a major event that causes numerous diseases. Recently, we systematically analyzed CD34^+^ cells in eight organs or tissues, including normal and diseased situations in mice and humans. They are mainly distributed in the fibroblast populations and have signal communications with other cells 40. In heart failure, it has been reported that CD34 cells can differentiate into fibroblasts and participate in cardiac fibrosis 14, 41, 42. Initially, we developed tdTomato and zsGREEN reporter genes to monitor CD34 and PI16 expression; however, *in vivo* experiments indicated that this tracking method was unsuccessful. We opted for the tdTomato reporter gene associated with the DT receptor as an alternative approach, enabling the selective ablation of CD34 and PI16 cells *in vivo*. Our *in vivo* study supports the ability of CD34^+^ PI16^+^ stem or progenitor cells to differentiate into various adventitial fibroblast subsets. This is the first evidence based on dual-recombinases lineage-tracing mouse models to demonstrate that fibroblast progenitor cells participate in reconstructing the adventitial microenvironment. CD34^+^ PI16^+^ progenitor cells can differentiate into different populations of fibroblasts, including myofibroblasts and cytokine-releasing fibroblasts in grafted vessels, contributing to vessel fibrosis and neointima lesion formation. It would be valuable to investigate the differentiation mechanism in the future, as this could help determine whether CD34^+^ PI16^+^ progenitor cells are uniquely present in all organs and play a role in fibrosis during disease progression.

Vascular fibrosis has been known to be critical for arterial remodeling 43. Based on the increased mass of the fibrotic cell layer in the adventitia, these cells could influence SMCs in the vessel wall after transplantation 44, with some factors potentially causing vessel remodeling 45. Moreover, numerous factors are reported to influence neointimal hyperplasia after injury 46-49. For instance, cytokine CCL11 was initially investigated in the context of asthma and tumors. However, recently, it has also been implicated in a spectrum of diseased conditions 50, 51. One of the major observations in the present study is that CD34^+^ PI16^+^ progenitor cells can become CD34^+^ PI16^-^ fibroblasts releasing CCL11. We used experimental methods such as protein chip screening, *in vivo* overexpression of CCL11 with AAV, and regulation of the specific receptor to demonstrate the heterogeneity of CD34^+^ cells and deleterious functions of CD34^+^ PI16^-^ cells. Overall, these results indicate that targeting different cell types, including fibroblast progenitor cell subsets and chemotactic fibroblast populations secreting CCL11, may influence the outcome of transplanted vessels.

The *in vivo* experiment involving the overexpression of CCL11 using AAV demonstrated that the green fluorescent protein tag associated with CCL11 was predominantly expressed in smooth muscle. However, findings from the dual-recombinase tracing experiment indicated a reduction in the hyperplasia of medial SMCs following the ablation of CD34 and PI16 cells. Additionally, fluorescence staining for CCL11 revealed its presence in both the media and adventitia. Considering that the serotype of the AAV is non-specific to particular cell types, a comprehensive assessment of the CCL11 source is warranted. Chemokines belonging to the CC group play important roles in the development of autoimmune and degenerative diseases, which have been well-documented 52-55. Generally, the CC chemokine receptors are coupled with Gαi/o. The effect of binding chemokines to their receptors inhibits AC, negatively affecting the levels of intracellular cAMP and the activation of PKA 56. Our study found that CCL11 released by fibroblasts can directly bind to CCR3 on SMCs of allografted vessels. Our data have indicated that binding of CCL11 to the CCR3 receptor leads to activating PI3K-AKT pathways in SMCs. A series of molecules from the PI3K-AKT signaling pathway are involved in smooth muscle cell migration to the intima, where they proliferate to form neointimal lesions in allografts. Therefore, fibrotic cells in the adventitia-released cytokines affect SMCs responsible for allograft vessel remodeling. Conversely, preventing chemokines from binding to receptor cells can serve therapeutic purposes.

Previous findings from our group have demonstrated that post-vascular injury, a subset of CD34 cells exhibits the ability to perform reparative functions within the endothelium 26, 57, 58. In the current study, we observed that the CD34^+^ PI16^+^ cells have the ability to differentiate into ECs, a phenomenon we observed but did not extensively investigate within the scope of this study. Further investigation into the molecular mechanisms that regulate the differentiation of CD34^+^ PI16^+^ cells into either ECs or fibroblasts would be highly valuable for clinical applications in cell therapy.

In summary, a combination of single-cell RNA sequencing and genetic cell lineage tracing techniques in this study revealed that bone marrow-derived CD34^+^ cells can differentiate into inflammatory cells. CD34^+^ PI16^+^ progenitor cells can also differentiate into a small population of ECs and different types of fibroblasts, in which CD34^+^ PI16^-^ fibroblasts can produce a large amount of chemokine CCL11. CCL11 can bind to CCR3 on SMCs, leading to cell migration to the intima, where they form neointima of allografted vessels. Blocking the binding of the chemokine significantly reduced neointimal lesions, indicating a therapeutic potential for transplant arteriosclerosis in the future.

## Supplementary Material

Supplementary methods and key resources table.

Supplementary figures 1-6.

Supplementary figures 7-8.

## Abbreviations

α-SMA: α-smooth muscle actin
DAPI: 4',6-Diamidino-2-Phenylindole
DT: diphtheria toxin
EC: endothelial cell
PCR: polymerase chain reaction
PDGFRα: platelet-derived growth factor receptor α
Sca-1: stem cells antigen-1
scRNA-seq: single-cell RNA sequencing
siRNA: small interfering ribonucleic acid
SMC: smooth muscle cell
CTGF: connective tissue growth factor
H&E: hematoxylin and eosin
adSPCs: adventitial stem or progenitor cells
CCL11: C-C Motif Chemokine Ligand 11
CCR3: C-C chemokine receptor type 3
