# Supplementary material for: CD34+ PI16+ fibroblast progenitors aggravate neointimal lesions of allograft arteries via CCL11/CCR3-PI3K/AKT pathway
Source: Theranostics. 2025 Jan 20;15(6):2523–43. doi: 10.7150/thno.104650 (PMC11840720; doi:10.7150/thno.104650)
Supplement: Supplementary file 1 — Supplementary methods and key resources table. [file thnov15p2523s1.pdf]

SUPPLEMENTARY MATERIALS

CD34$^+$/PI16$^+$ fibroblast progenitors aggravate neointimal lesions of allograft arteries via CCL11/CCR3-PI3K/AKT Pathway

Supplementary methods

Key resources table

SUPPLEMENTARY METHODS

Mice Breeding and Genotyping

All animal procedures conformed to the Guide for Care and Use of Laboratory Animals published by the US National Institute of Health (8th edition, 2011) and were approved by the Institutional Animal Care and Use Committee of Zhejiang University School of Medicine. The CD34-CreERT2 and PI16-CreERT2 knock-in mouse models were independently engineered by Shanghai Model Organisms Center, Inc. and GemPharmatech Inc. The targeting strategy involved inserting a P2A-CreERT2 cassette immediately downstream of the last exon, before the 3’ UTR, of each target gene. The CRISPR/Cas9 system was employed to introduce this cassette into the mouse genome on a C57BL/6J background. Vectors containing the sgRNA and Cas9 mRNA were synthesized in vitro and then microinjected into fertilized C57BL/6J oocytes. Homologous recombination in the F0 generation was confirmed by extended PCR spanning the homologous arms, with sequences of the PCR products validated by sequencing. The F0 founders were then bred with C57BL/6J wild-type mice to generate heterozygous CD34-CreERT2 and PI16-CreERT2 progeny.C57BL/6-Gt (ROSA)26Sortm1 (HBEGF)Awai/J, C57BL/6Smoc-Gt (ROSA)26Sorem1(CAG-LSL-RSR-tdTomato-2ADTR) Smoc, B6. Cg -Gt (ROSA) 26Sortm9(CAGtdTomato)Hze/J as well as Balbc and C57BL/6J mice lines were purchased from Shanghai Model Organisms Center, Inc. Male and female mice aged 8-12 weeks old were utilized and evenly distributed across various experimental cohorts, with group sizes specified in the associated figure legends. The mice were provided with a standard lab chow, unrestricted access to food and water, and housed in an environment maintained at a temperature of 22 ± 1°C with 65–70% humidity under a consistent 12-hour light-dark cycle. All mice, whether bred in-house or acquired, were acclimatized at the Zhejiang University Experimental Animal Center (Hangzhou, China) for a minimum of one week prior to the commencement of experiments. The animal cohort size was determined a priori, grounded on data from preliminary studies, targeting a significance level (alpha) of 0.05 and a statistical power (1-beta) of 0.8. All animal procedures were replicated a minimum of three times to ensure the robustness of the findings. In instances of allogeneic vascular transplantation, mice were humanely euthanized if deemed clinically necessary due to deteriorating health. To mitigate issues of sample interdependence, mice sourced from external suppliers were selected from varied litters throughout the three-year investigation

Establishment of mouse transplant arteriosclerosis model

Donor preparation: use 8 to10 week-old Balb/c mice, anesthetize, dissect, and expose the thoracic aorta, infuse heparinized saline (200UI/ml), and use an electro coagulator to carefully cauterize each intercostal space artery, and cut the thoracic aorta and store it in 4°C physiological saline for subsequent use. Arterial transplant: twelve-week-old C57 mice were anesthetized with 3.0 Vol% isoflurane (RWD Life Science Inc., Cat#: R510-22-10) and maintained at 1-2% in a general anesthesia chamber. After securing the mouse, the neck area was shaved and sterilized. An incision was made to expose the connective tissue and glands, followed by the use of electrocoagulation to cauterize the right sternocleidomastoid

muscle, revealing the right carotid artery segment. Two 10-0 nylon sutures were placed side by side on the carotid artery within the designated region. The recipient's carotid artery was then excised between the sutures, and lifted by the threads to facilitate the insertion of a cannula, which was then secured onto the artery. An arterial clamp was applied to the extension of the cannula, anchored to the right neck dermis, and tied off. The vessel was flushed with heparinized saline below the clamp to clear the lumen, and the vessel wall was carefully everted over the cannula. The donor's vessel was aligned with the blood flow direction over the severed carotid artery ends and securely sutured with 10-0 silk. Upon releasing the clamps at both ends, restoration of blood flow and vessel pulsation were observed. Transplant recipient mice were fed increased jelly-containing NSAIDs during the perioperative period. Graft collection: Following the transplantation at specified intervals, mice were humanely euthanized utilizing carbon dioxide (CO2) in alignment with the NIH Guidelines for animal euthanasia, ensuring minimal distress. In brief, a cohort of 3-5 mice was placed inside a 20-liter capacity euthanasia chamber. A cylinder emitting 99.99% pure CO2 was linked to the chamber, dispensing the gas at a flow rate of 10 liters per minute. Within 3 minutes, the mice became unconscious, evidenced by the absence of spontaneous respiratory movements. An additional minute of CO2 flow ensured complete cessation of breathing; this was verified by observing faded eye color and no reaction of the pupils to light. Subsequently, each mouse was removed from the chamber, underwent thoracotomy, and the heart was perfused via left ventricular puncture with PBS. The vascular grafts, whether allografts or isografts, were then excised for further analysis. Death was conclusively ascertained following cardiac excision.

Diphtheria toxin induces lineage cell apoptosis

CD34CreERT2; R26DTR and CD34Dre PI16CreERT2; R26DTR mice underwent intraperitoneal injections of diphtheria toxin (DT) at a dosage of 5ng/g daily over five days. Three weeks post-injection, the efficacy of DT-mediated cell ablation was assessed. This allowed for the evaluation of vascular remodeling in the mice, examining the role of $CD34^+$ or $CD34^+$ $PI16^+$ stem/progenitor cells in vascular reconstruction following transplantation and assessing the effects of induced apoptosis on vascular architecture.

Magnetic bead sorting of CD34-positive cells

The adventitia was carefully removed from the transplanted vessel, sectioned into 1-2mm pieces, and immersed in DMEM supplemented with 20% FBS for 20 minutes. These tissue fragments were then adhered to a T25 culture dish pre-coated with a suitable substrate and incubated at 37°C in a 5% CO2 atmosphere for 2-4 hours before the introduction of a complete growth medium composed of DMEM, 20% FBS, penicillin-streptomycin (1X), 50nM beta-mercaptoethanol, and 10ng/ml mouse leukemia inhibitory factor. The medium was refreshed every two to three days. Following 3 to 5 passages, cells were dissociated using 0.25% trypsin-EDTA, and CD34 positive and negative fibroblasts were subsequently segregated employing magnetic bead separation specific to CD34.

Flow cytometric analysis

Isolated cells from allograft and aorta were incubated with red blood cell lysis buffer to lyse red blood cells and filtered through a 40μm cell strainer to obtain single-cell suspensions. For staining certain cell surface markers, cells were stained with conjugated antibodies (1 μg per 106 cells) at 4°C for 30 minutes. Antibodies used included CD34-FITC (BD pharmingen, 555821), CD31-APC (BD pharmingen, 50994), CD140a (Invitrogen,17-1401-81), CD45-PerCP-Cy5.5 (BD pharmingen, 555821) and aSMA-FITC (Sigma, F3777). After staining, cells were washed, re-suspended in PBS, and stained for 20 minutes with the LIVE/DEAD™ Fixable Near-IR Dead Cell Stain Kit (Invitrogen, L34975, 1:1000) and Hoechst 33342 (Invitrogen, H3570, 1:1000) to exclude designated dead cells from the analysis. Cells were then washed again and re-suspended in PBS containing 0.1% FBS for flow cytometric analysis. All prepared samples were analyzed on a BD LSR Fortessa II flow cytometer (BD Biosciences). Flow cytometry data were analyzed with FlowJo v10 software (BD Biosciences, USA).

Flow cytometric sorting and single-cell RNA sequencing

Four weeks post-operation, allografts from each experimental group were harvested from six male and female CD34CreERT2; Rosa26-tdTomato mice. Grafts were rinsed three times in PBS, finely minced, and enzymatically dissociated in a shaking water bath at 37°C with a digestion cocktail comprising papain, collagenase I, and dispase II as delineated in prior protocols. Cells liberated into the supernatant were harvested and suspended in DMEM with 10% FBS. Cells from each graft were pooled following digestion completion. Following adequate dissociation, the cell suspension was filtered through a 40-μm mesh to remove undigested debris and then centrifuged for 8 minutes at 500g at 4°C. Single cells were collected, washed with PBS, resuspended in 0.04% BSA, and tdtomato-positive single mononuclear live cells (Hoechst positive and dead cell stain negative) were sorted using the BD FACS ARIA II flow cytometer (BD Biosciences). The sorted cells were then subjected to scRNA-seq. Libraries were prepared using the Chromium TM Single Cell 3' Reagent Kit v3 chemistry (10x Genomics) following the standard protocol. Libraries were sequenced on the Novaseq6000 PE150 platform (Illumina) with a paired-end 150 bp sequencing strategy. The 10x Chromium TM processes, library generation, and sequencing were performed by Novogene Co., Ltd (Beijing, China).

Chemokine protein microarray detection of cell supernatants

The cell supernatant of the target cell sample was tested by mouse chemokine array (QAM-CHE-1, RayBiotech) according to the manufacturer's instructions. CD34-positive and CD34-negative cells with good growth status were inoculated in 6-well plates respectively. After 12 hours of serum-free culture, the cell culture medium was collected and centrifuged at 2000r/min at 4 degrees for 15 minutes. The target cell supernatant was collected for further detection. Next, the antibody slide was processed. Do not touch the slide, block the cytokine antibody on the slide, and incubate at 4 degrees overnight. Then 100ul of the sample was added to each well, incubated at room temperature for 1 hour, and washed 3 times; then the

antibody-containing bioluminescent was added, incubated at room temperature for 1 hour, and washed 3 times. All slides were scanned using GenePix 4000 B chip scanner and analyzed using GenePix Pro 6.0 software. The protein level was normalized with the internal reference, and the expression of each factor was analyzed according to the fluorescence intensity to screen out valuable secreted proteins.

Western blot analysis

Cultured cells or vascular isografts were lysed in RIPA lysis buffer supplemented with phosphatase inhibitor tablets (Roche, 04906837001) and protease inhibitor tablets (Roche, 05892970001). Protein concentration was quantified by an enhanced BCA protein assay kit (Beyotime) according to the manufacturer's instructions. Equal amounts of total protein lysates from each sample were subjected to electrophoresis on SDS polyacrylamide gels (Bio-Rad), followed by transfer onto PVDF membranes (Millipore) for standard Western blotting procedures. After blocking in 5% milk at room temperature for 1 hour, the PVDF membranes were incubated with primary antibodies overnight at 4°C, followed by the appropriate secondary antibodies at room temperature for 60 minutes. Membranes were detected using an enhanced chemiluminescence (ECL) method and images were captured with a bio-spectral imaging system (UVP, Upland, CA). Primary antibodies used in this study included CD34(Santa Cruz, SC-7324), PI16(R&D, AF4929), PDGFRa(CST,3174), PDGFRb(CST,3169), Periostin(R&D,AF2955), Vimentin(Santa Cruz,SC-6260), PI3K-P110(CST, 4249), PI3K-P85(CST,4292), AKT(CST,9272), p-AKT(CST,4060), MMP2(Huabio, ET1606-4), MMP9(Santa Cruz,SC-21733), SMA(Proteintech, 23081-1-AP), SM22(ABCAM,AB14106), TUBULIN(Huabio,ET1702-68), ACTIN(Huabio, ET1702-67), and GAPDH(Huabio, HA721131).

Transwell migration assay

Transwell migration assays were performed using Transwell cell culture plates (Corning Costar, 3422) with 6.5 µm pore size filters. Smooth muscle cells (1 × 106 cells/100 µl serum-free medium) were seeded in the upper chamber, and then CD34+ cells and CD34- cells were added to the lower chamber, respectively. For CCL11 overexpression and CCR3 knockdown experiments, smooth muscle cells were added to the upper chamber. No stimulation was added to the control group, and CCL11 overexpression plasmid and CCR3-siRNA spike were used in the experimental group. After 24 hours of incubation, remove the chamber and use a cotton swab to remove non-migrated cells remaining on the top of the Transwell filter. Migrated cells on the lower surface of the Transwell filter were fixed in 4% PFA for 10 min and then stained with 1% crystal violet (Sigma, HT90132) for 15 min. Images were acquired using a Nikon Eclipse TS100 microscope. Count cells in 5 random fields under a microscope.

Scratch-wound healing assay

Cells (1 × 105 cells per well) were seeded into 6-well plates and maintained in a complete medium. Once cells reach 80% confluence, create a wound by scraping the well from top to

bottom using a 1 mL pipette tip. Take random images of scratches in 6 fields of view under a microscope. After 24h of incubation, images of the scratch wound were again acquired in 6 random fields. Data shown are relative averages of cells migrating to the scratch wound area at the 24h time point compared to the initial scratch wound.

ELISA assay of cell supernatants

The levels of CCL3(MultiSciences, EK261), CCL5(MultiSciences, EK2129), CCL11(MultiSciences, EK2130), CCL12(Biocompare, MBS825064), CXCL2(MultiSciences, EK2142), CXCL5(ABCAM, AB264611) and CXCL12(Biocompare, EM0174) in cell culture supernatants were detected by mouse chemokine ELISA kit. All experiments were performed according to the standard protocol provided by the manufacturer.

CCK8 proliferation assay

Seed the cells in a 96-well plate, with about 100 μl (about 5,000 cells) per well, and incubate it in a 37°C, 5% CO2 incubator. After the cells adhere, add 10 μl CCK-8 solution to each well, place the culture plate into Incubate in the incubator for 24 hours, and measure the absorbance value (OD) at 450 nm with a microplate reader.

Key resources table

REAGENT or RESOURCE	SOURCE	IDENTIFIER
**Antibodies**		
CD34	Abcam	Cat#AB81289; RRID: AB_1640331
CD31	R&D	Cat#AF3628； RRID: AB_2161028
aSMA-FITC	Sigma-Aidrich	Cat#F3777; RRID:AB_476977
PDGFRa	R&D	Cat#AF1062; RRID:AB_2236897
CD45	R&D	Cat#AF114; RRID: AB_442146
CD34-FITC	BD pharmingen	Cat#555821; RRID: AB_396150
CD31-APC	BD pharmingen	Cat#561814; RRID: AB_10893351
CD34-APC	BD pharmingen	Cat#555824; RRID: AB_398614
CD140a-APC	Invitrogen	Cat#17-1401-81; RRID: AB_529482
CD45-PerCP-Cy5.5	BD pharmingen	Cat#550994; RRID: AB_394003
Periostin	R&D	Cat#AF2955; RRID: AB_664123
Vimentin	Abcam	Cat#ab8978; RRID: AB_306907
PDGF Receptor a	CST	Cat#3174; RRID: AB_2162345
PDGF Receptor β	CST	Cat#3169; RRID: AB_2162497
Vimentin	Santa Cruz	Cat#sc-6260; RRID: AB_628437
CD34	Santa Cruz	Cat#sc-7324; RRID: AB_2291280
GAPDH-HRP	Huabio	Cat#HA721131; RRID: AB_3072255
PI16	R&D	Cat#AF4929; RRID: AB_2299601
tdTomato	Rockland	Cat#600-401-379; RRID: AB_2209751

tdTomato	SICGEN	Cat#AB8181-200;<br>RRID: AB_2722750
GFP	Abcam	Cat# ab6662；<br>RRID:AB_305635
CCL11	R&D	Cat#AF-420-NA;<br>RRID: AB_354486
Ki-67	Abcam	Cat#ab16667；<br>RRID: AB_302459
CCR3	Proteintech	Cat#22351-1-AP;<br>RRID: AB_2879085
PI3 Kinase p110α	CST	Cat#4249;<br>RRID: AB_2165248
PI3 Kinase p85	CST	Cat#4292;<br>RRID: AB_329869
Phospho-Akt (Ser473)	CST	Cat#4060;<br>RRID: AB_2315049
Akt	CST	Cat#9272;<br>RRID: AB_329827
MMP2	Huabio	Cat#ET1606-4;<br>RRID: AB_3069735
MMP9	Santa Cruz	Cat#sc-21733;<br>RRID: AB_627959
Actin-HRP	Huabio	Cat#ET1702-67;<br>RRID: AB_2890197
SM22	Abcam	Cat#AB14106;<br>RRID: AB_443021
Tubulin	Huabio	Cat#ET1702-68;<br>RRID: AB_3070328
SMA	Proteintech	Cat#23081-1-AP;<br>RRID: AB_2815024
OPN	R&D	Cat# AF808;<br>RRID:AB_2194992
Bacterial and virus strains		
pAAV-CMV-MCS-EF1-mNeonGreen-WPRE	OBiO Tech, Inc	H12049
pAAV-CMV-Ccl11-3xFLAG-EF1-mNeonGreen-WPRE	OBiO Tech, Inc	H24912
Biological samples		
Chemicals, peptides, and recombinant proteins		
DMEM	ATCC	302002
RPMI 1640	Biological industry	01-100-1A

Papain	Sigma	P4762
1,4-Dithioerythritol	Sigma	D8255
Taurine	Sigma	T8691
Collagenase I	Gibco	17018029
Dispase II	Sigma	D4693
HBSS	Gibco	14025092
O.C.T	Sakura	4583
DAPI	Servicebio	G1012
Triton X-100	Sigma	T8787
FBS	Gibco	10099141C
LIF	Merck millipore	LIF1050
Penicillin-Streptomycin	Gibco	21985-023
Gelatin	Sigma	G1393
TRIzol™	Thermo fisher scientific	15596018
LIVE/DEAD™ Fixable Near-IR Dead Cell Stain Kit	Invitrogen	L34975
Hoechst 33342	Invitrogen	H3570
Tamoxifen	Sigma	T5648
Corn oil	Sigma	C8267
Diphtheria toxin	Sigma	D0564
PFA	Servicebio	G1101
CCL11	R&D	AF-420-NA
CCL11	Abclonal	RP01635
Rosmarinic acid	Selleck	S3612
MK-2206 2HCl	Selleck	S1078
CCL11-Plasmid	Shanghai Zorin Biotech	N/A
Critical commercial assays		
Quantibody® Mouse Chemokine Array	Raybiotech	Catalog #: QAM-CHE-1
Mouse CCL3/MIP-1α ELISA Kit	MultiSciences	EK261
Mouse CCL5/RANTES ELISA Kit	MultiSciences	EK2129
Mouse CCL11/Eotaxin ELISA Kit	MultiSciences	EK2130
Mouse CCL12/MCP-5 ELISA Kit	Biocompare	MBS825064
Mouse CXCL2/MIP-2 ELISA Kit	MultiSciences	EK2142
Mouse CXCL5/LIX ELISA Kit	ABCAM	AB264611
Mouse CXCL12/SDF-1 ELISA Kit	Biocompare	EM0174

Deposited data		
Bulk-RNA sequencing	uploading	
Single-cell RNA sequencing	uploading	
Single cell RNA sequencing	(Chen et al., 2023)	GSE211731
Experimental models: Cell lines		
MOVAS	ATCC	CRL-2797
Experimental models: Organisms/strains		
CD34-CreERT^2	Shanghai Biomodel Organism	Self-designed
CD34-Dre	Shanghai Biomodel Organism	Self-designed
PI16-CreERT^2	GemPharmatech	T057690
C57BL/6-Gt(ROSA)26Sortm1(HBEGF)Awai/J	Shanghai Biomodel Organism	JAX: 007900
C57BL/6Smoc-*Gt(ROSA)26Sorem1(CAG-LSL-RSR-tdTomato-2A-DTR)Smoc*	Shanghai Biomodel Organism	NM-KI-190086
B6.Cg-Gt(ROSA)26Sortm9(CAGtdTomato)Hze/J	Shanghai Biomodel Organism	JAX: 007909
C57BL/6J	Shanghai Biomodel Organism	JAX: 000664
BALB/cAnSmoc	Shanghai Biomodel Organism	SM-003
Oligonucleotides		
Recombinant DNA		
Software and algorithms		
GraphPad Prism 6 software	GraphPad Software, Inc.	N/A
FlowJo software	Tree Star, Ashland, Ore	N/A
Cell Ranger (v.2.1.1)	10X Genomics	https://www.10xgenomics.com/support
Seurat v2.3	R. Satija Lab	https://satijalab.org/seurat/

Other		
