# Supplementary material for: CD34+ PI16+ fibroblast progenitors aggravate neointimal lesions of allograft arteries via CCL11/CCR3-PI3K/AKT pathway
Source: Theranostics. 2025 Jan 20;15(6):2523–43. doi: 10.7150/thno.104650 (PMC11840720; doi:10.7150/thno.104650)
Supplement: Supplementary file 2 — Supplementary figures 1-6. [file thnov15p2523s2.pdf]

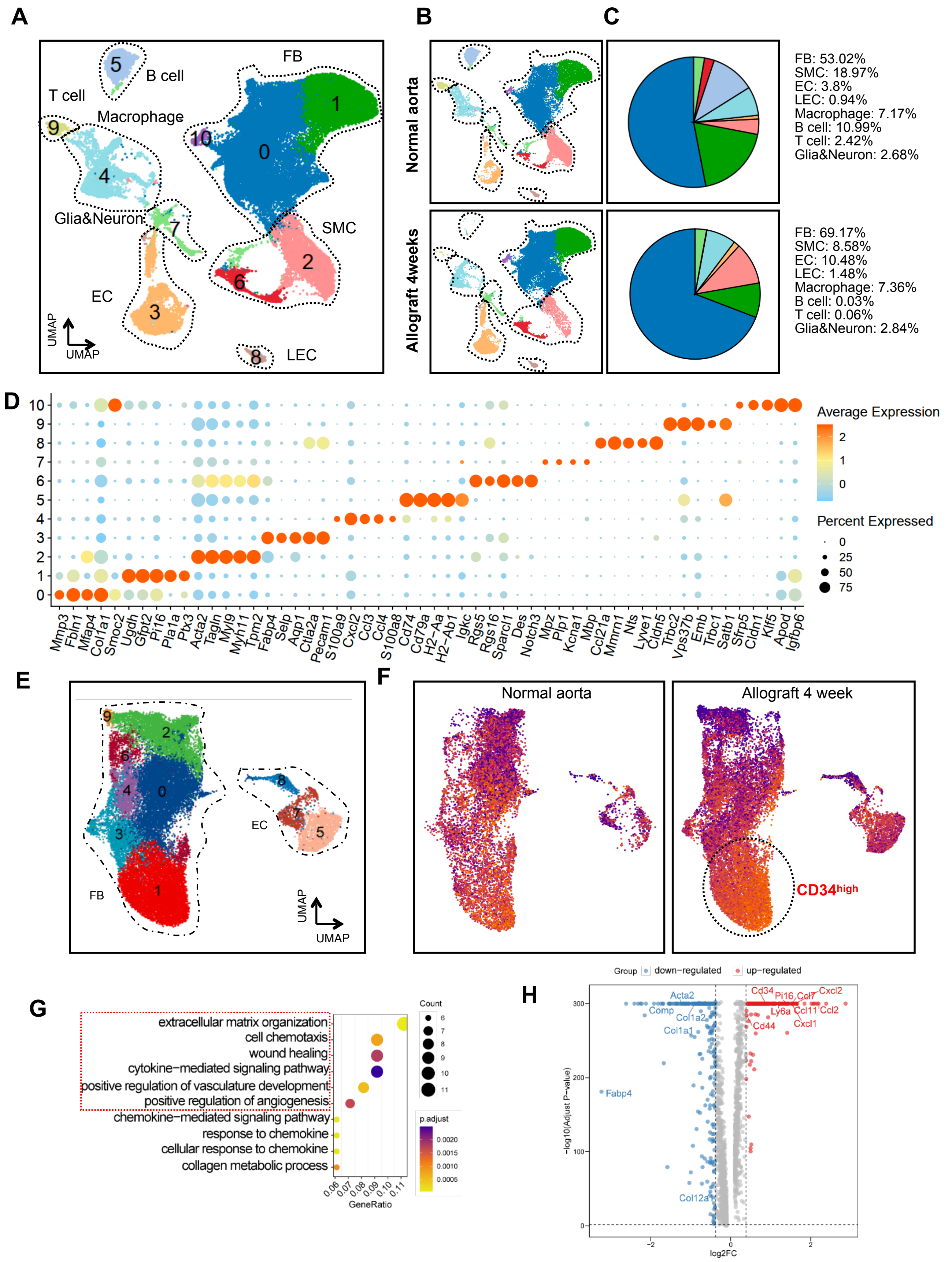
A
B
C
FB
B cell
T cell
Macrophage
Glia&Neuron
SMC
EC
LEC
UMAP
UMAP
Normal aorta
Allograft 4weeks
FB: 53.02%
SMC: 18.97%
EC: 3.8%
LEC: 0.94%
Macrophage: 7.17%
B cell: 10.99%
T cell: 2.42%
Glia&Neuron: 2.68%
FB: 69.17%
SMC: 8.58%
EC: 10.48%
LEC: 1.48%
Macrophage: 7.36%
B cell: 0.03%
T cell: 0.06%
Glia&Neuron: 2.84%
D
Average Expression
Percent Expressed
Mmp3 Fbln1 Mfap4 Col1a1 Smoc2 Ugdh Gfpt2 Pi16 Pla1a Ptx3 Acta2 Tagln Myl9 Myh11 Tpm2 Fabp4 Selp Aqp1 Ctla2a Pecam1 S100a9 Cxcl2 Ccl3 Ccl4 S100a8 Cd74 Cd79a H2-Aa H2-Ab1 Igkc Rgs5 Rgs16 Sparcl1 Des Notch3 Mpz Plp1 Kcna1 Mbp Ccl21a Mmrn1 Nts Lyve1 Cldn5 Trbc2 Vps37b Emb Trbc1 Satb1 Sfrp5 Cldn1 Klf5 Apod Igfbp6
E
FB
EC
UMAP
UMAP
F
Normal aorta
Allograft 4 week
CD34high
G
extracellular matrix organization
cell chemotaxis
wound healing
cytokine−mediated signaling pathway
positive regulation of vasculature development
positive regulation of angiogenesis
chemokine−mediated signaling pathway
response to chemokine
cellular response to chemokine
collagen metabolic process
GeneRatio
Count
p.adjust
H
Group down−regulated up−regulated
Acta2
Comp
Col1a2
Col1a1
Fabp4
Col12a1
Cd34
Pi16
Ccl7
Cxcl2
Ly6a
Ccl11
Ccl2
Cd44
Cxcl1
−log10(Adjust P−value)
log2FC

Figure S1 (Related to Figure 1). Single-cell RNA-sequencing analysis of Cellular Component and CD34 heterogeneity.

(A) UMAP plots showing 11 color-coded cell clusters in the normal thoracic aortas and grafted arteries (n = 43178 cells) and clusters are identified via biological specific markers. (B) UMAP plots showing color-coded cell clusters in either normal thoracic aortas (19400 cells) or 4-week grafted arteries (23778 cells). (C) Pie chart showing the proportion of major cell types among different groups in either normal thoracic aortas or 4-week grafted arteries. (D) Dot plot showing average scaled expression levels of top DEGs across identified cell clusters. Dot size reflects the percentage of cells expressing the selected gene in each cell cluster. (E) UMAP plots showing the major cell types and 10 color-coded cell clusters of endothelial cells and fibroblasts in the normal thoracic aortas and grafted arteries. (F) Feature plots showing that CD34 expression levels in either normal thoracic aortas or 4-week grafted arteries. (G) Gene ontology analysis of the top enriched biological function for cluster 1. (H) Volcano plots showing the up-regulated and down-regulated gene for cluster 1 compared to other fibroblast clusters.

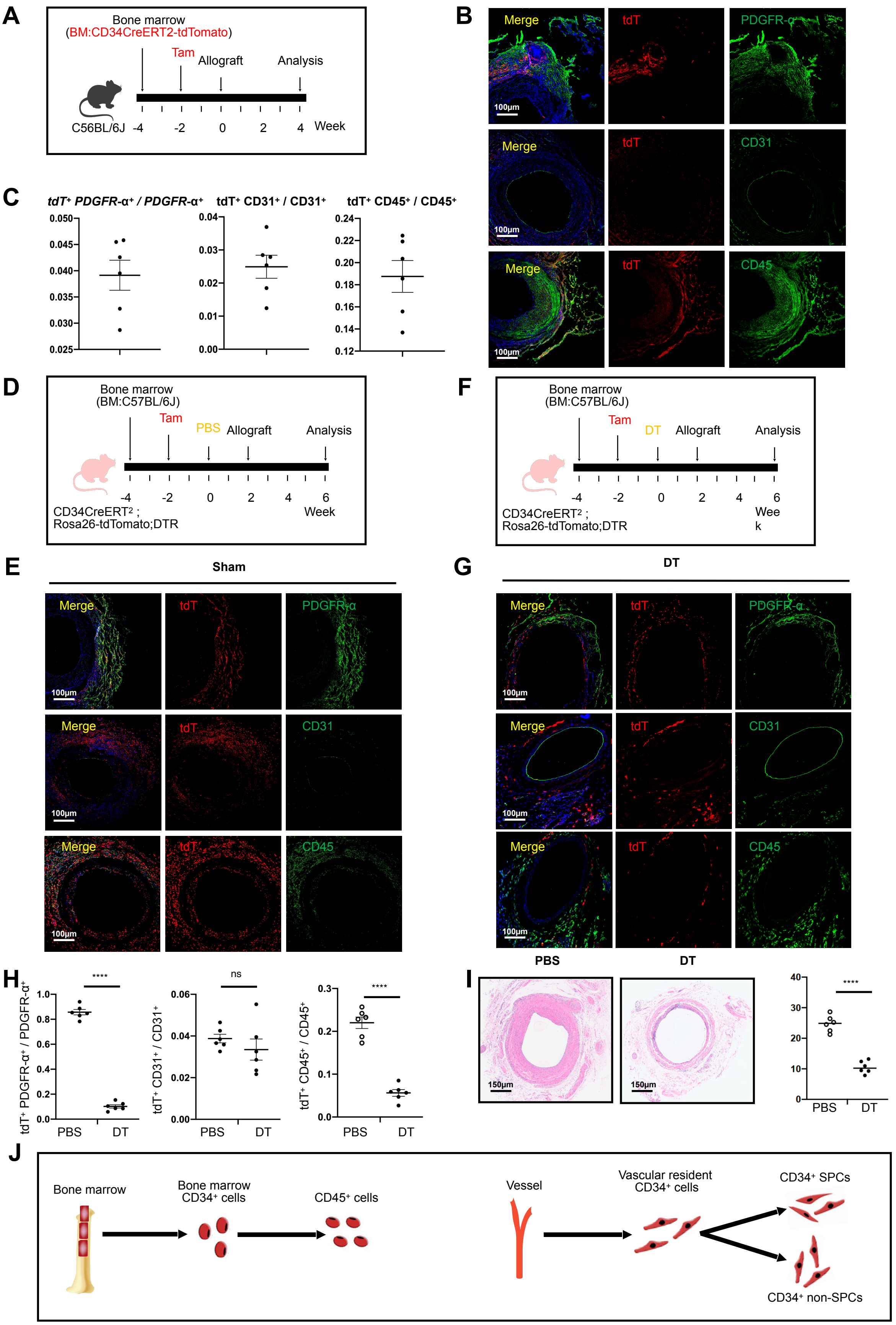
A
Bone marrow
(BM:CD34CreERT2-tdTomato)
Tam
Allograft
Analysis
C56BL/6J
-4 -2 0 2 4 Week
B
Merge
tdT
PDGFR-α
CD31
CD45
100μm
C
tdT+ PDGFR-α+ / PDGFR-α+
tdT+ CD31+ / CD31+
tdT+ CD45+ / CD45+
D
Bone marrow
(BM:C57BL/6J)
Tam
PBS
Allograft
Analysis
CD34CreERT2 ;
Rosa26-tdTomato;DTR
-4 -2 0 2 4 6 Week
F
Bone marrow
(BM:C57BL/6J)
Tam
DT
Allograft
Analysis
CD34CreERT2 ;
Rosa26-tdTomato;DTR
E
Sham
G
DT
H
tdT+ PDGFR-α+ / PDGFR-α+
tdT+ CD31+ / CD31+
tdT+ CD45+ / CD45+
PBS
DT
****
ns
I
PBS
DT
150μm
J
Bone marrow
Bone marrow
CD34+ cells
CD45+ cells
Vessel
Vascular resident
CD34+ cells
CD34+ SPCs
CD34+ non-SPCs

Figure S2 (Related to Figure 2). Effect of bone marrow-derived and non-bone marrow-derived CD34⁺ cells on vascular remodeling.
(A/D/F) Sketch of the experimental strategy for bone marrow transplant chimeric mice model. (B) Representative co-immunofluorescence images of tdTomato and PDGFR-α, CD31, and CD45 on cryosections from 4 weeks grafted arteries of chimeric mice (n = 6). (C) semi-quantification of the percentage of $tdTomato^+$ cells that express PDGFR-α, CD31, and CD45 in (B). Data are presented as mean ± SEM. (E) Representative co-immunofluorescence images of tdTomato and PDGFR-α, CD31, and CD45 on cryosections from 4-week grafted arteries of chimeric mice without DT treatment (n = 6). (G) Representative co-immunofluorescence images of tdTomato and PDGFR-α, CD31, and CD45 on cryosections from 4-week grafted arteries of chimeric mice with DT treatment (n = 6). (H) semi-quantification of the percentage of $tdTomato^+$ cells that express PDGFR-α, CD31, and CD45 in (E and G). Data were presented as the mean ± SEM and analyzed by using an unpaired two-tailed Student's t-test. (I) Representative HE staining of grafted arteries with or without DT treatment (n = 6); and semi-quantification of neointimal area in the presence and ablation of $CD34^+$ cells. Data were presented as the mean ± SEM and analyzed by using an unpaired two-tailed Student's t-test. * $p < 0.05$, ** $p < 0.01$, *** $p < 0.001$, **** $p < 0.0001$. (J) Cartoon image unveiling the heterogeneity of $CD34^+$ cells.

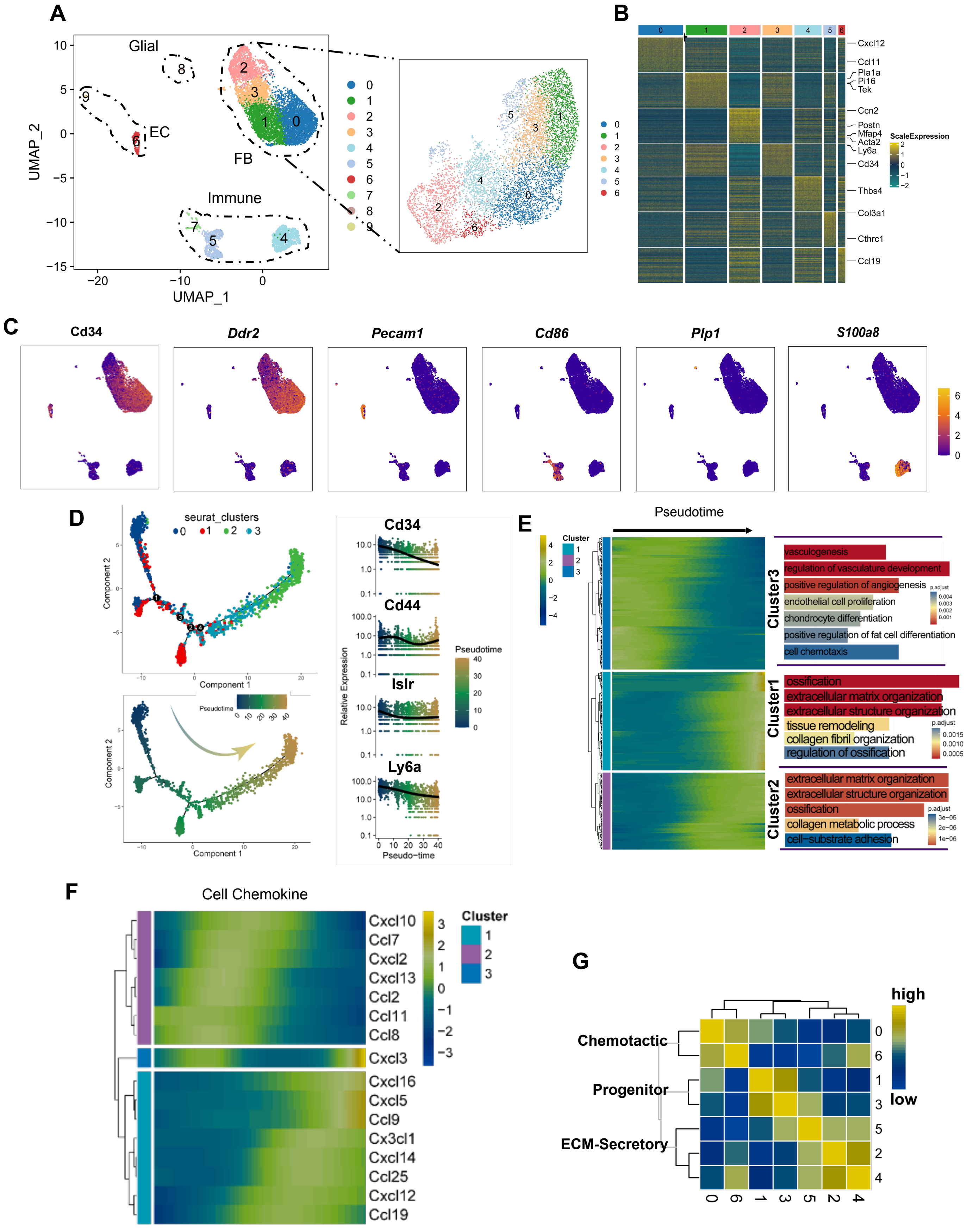
A
Glial
EC
FB
Immune
UMAP_2
UMAP_1
B
Cxcl12
Ccl11
Pla1a
Pi16
Tek
Ccn2
Postn
Mfap4
Acta2
Ly6a
Cd34
Thbs4
Col3a1
Cthrc1
Ccl19
ScaleExpression
C
Cd34
Ddr2
Pecam1
Cd86
Plp1
S100a8
D
seurat_clusters
Component 2
Component 1
Pseudotime
Cd34
Cd44
Islr
Ly6a
Relative Expression
Pseudo-time
E
Pseudotime
Cluster
Cluster3
vasculogenesis
regulation of vasculature development
positive regulation of angiogenesis
endothelial cell proliferation
chondrocyte differentiation
positive regulation of fat cell differentiation
cell chemotaxis
Cluster1
ossification
extracellular matrix organization
extracellular structure organization
tissue remodeling
collagen fibril organization
regulation of ossification
Cluster2
extracellular matrix organization
extracellular structure organization
ossification
collagen metabolic process
cell−substrate adhesion
p.adjust
F
Cell Chemokine
Cxcl10
Ccl7
Cxcl2
Cxcl13
Ccl2
Ccl11
Ccl8
Cxcl3
Cxcl16
Cxcl5
Ccl9
Cx3cl1
Cxcl14
Ccl25
Cxcl12
Ccl19
Cluster
G
Chemotactic
Progenitor
ECM-Secretory
high
low

Figure S3 (Related to Figure 2). Single-cell RNA-sequencing analysis of tdTomato^{+} cells labeled by CD34 lineage tracing technique.
(A) UMAP plots showing the major cell types and 10 color-coded cell clusters selected from tdTomato+ cells in the aortic graft. n = 9299 cells. (B) Heatmap showing average scaled expression levels of top DEGs across identified cell clusters. (C) Feature plots show that these markers expression (Cd34/Ddr2/Pecam1/Cd86/
Plp1/S100a8) levels of tdTomato+ cells in the aortic graft. (D) Pseudotime heatmap showing top enriched functions in clusters1-3. (E) Heatmap showing the top expressing genes of cell chemokine in clusters1-3. (F) Trajectory plot showing the pseudotime representing trajectories of CD34-high expressing cells differentiation and the expression of 4 markers (CD34/CD44/Islr/Ly6a) in the differentiation trajectory. (G) Heatmap analysis for the different functions of fibroblasts after transplantation.

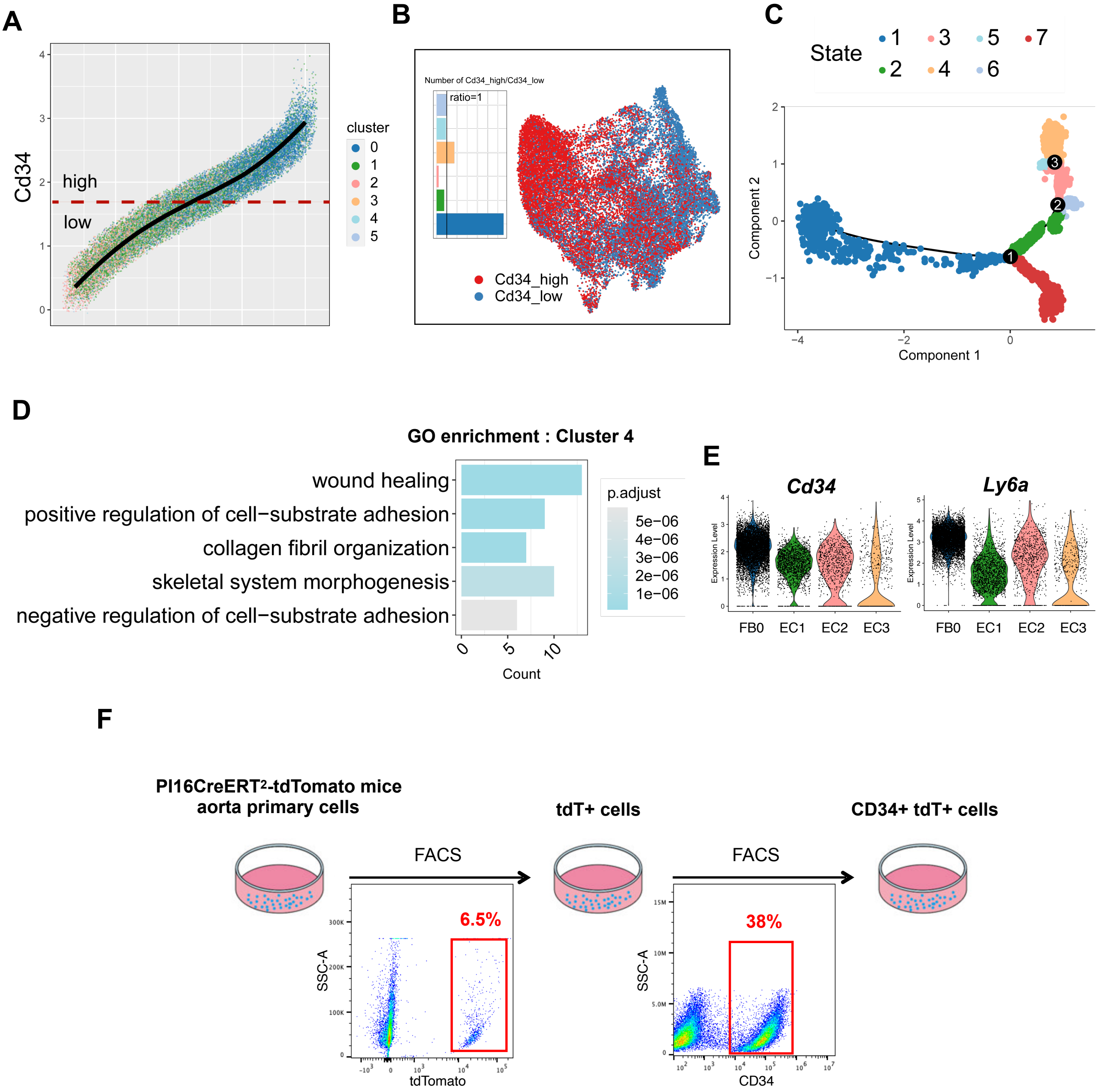

Figure S4 (Related to Figure 3). Single-cell sequencing analysis of CD34⁺ PI16⁺ SPC subpopulation.
(A) Feature plots showing the dividing line between high and low expression of CD34. (B) UMAP plots showing CD34 expression levels in each cluster. (C) Pseudotime trajectories showing the seven states of CD34 and PI16-high expressing cells differentiation. (D) Gene Ontology analysis of top enriched functions in cluster 4. (E) The expression of CD34 and Ly6a in cluster 0. (F) Schematic diagram of cell sorting process and flow cytometric sorting chart of CD34 and Pi16 positive cells.

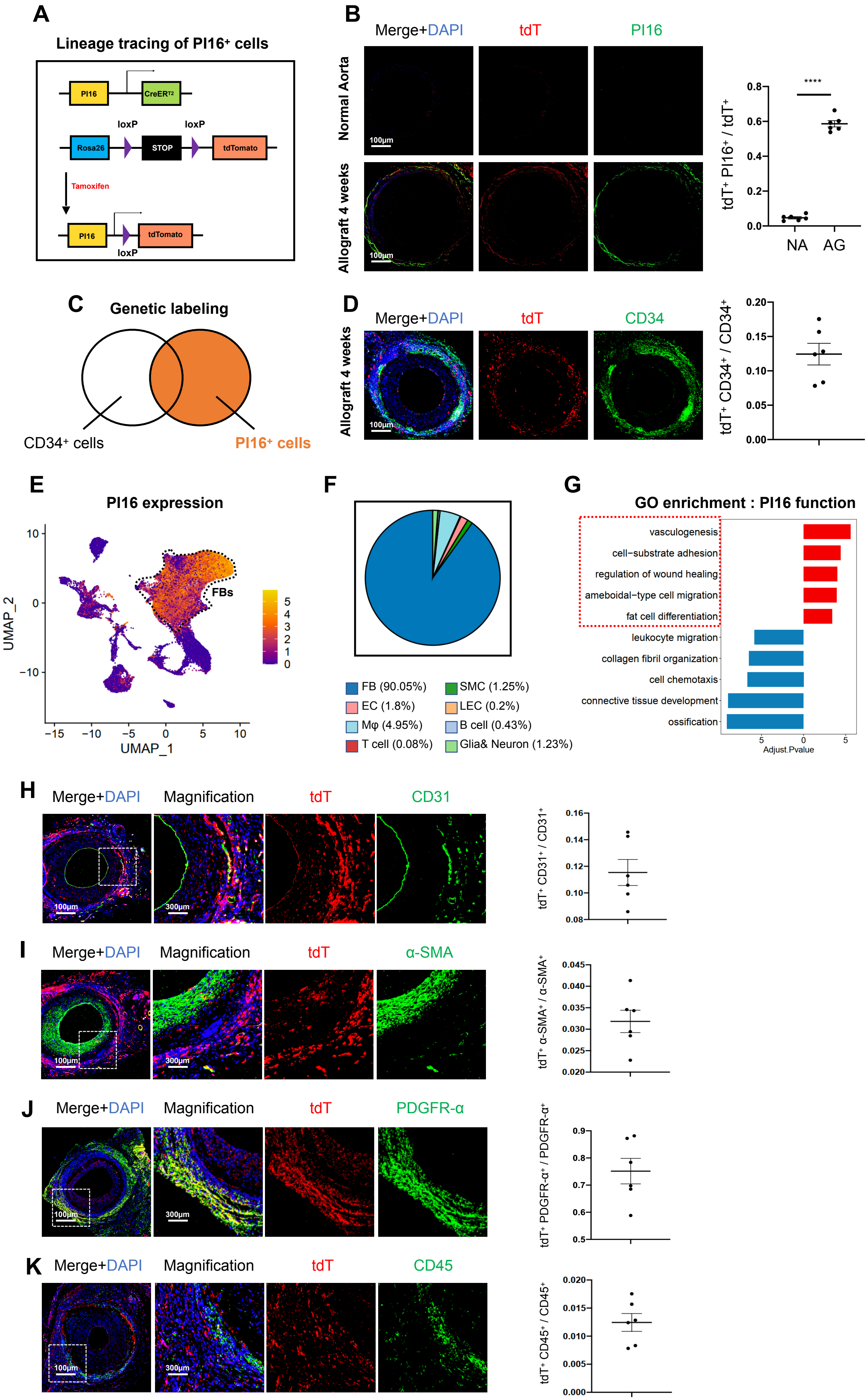

A
Lineage tracing of PI16+ cells
PI16
CreERT2
loxP
loxP
Rosa26
STOP
tdTomato
Tamoxifen
PI16
tdTomato
loxP
B
Merge+DAPI
tdT
PI16
Normal Aorta
Allograft 4 weeks
100μm
tdT+ PI16+ / tdT+
****
NA
AG
C
Genetic labeling
CD34+ cells
PI16+ cells
D
Merge+DAPI
tdT
CD34
Allograft 4 weeks
tdT+ CD34+ / CD34+
E
PI16 expression
FBs
UMAP_1
UMAP_2
F
FB (90.05%)
SMC (1.25%)
EC (1.8%)
LEC (0.2%)
Mφ (4.95%)
B cell (0.43%)
T cell (0.08%)
Glia& Neuron (1.23%)
G
GO enrichment : PI16 function
vasculogenesis
cell−substrate adhesion
regulation of wound healing
ameboidal−type cell migration
fat cell differentiation
leukocyte migration
collagen fibril organization
cell chemotaxis
connective tissue development
ossification
Adjust.Pvalue
H
Merge+DAPI
Magnification
tdT
CD31
300μm
tdT+ CD31+ / CD31+
I
α-SMA
tdT+ α-SMA+ / α-SMA+
J
PDGFR-α
tdT+ PDGFR-α+ / PDGFR-α+
K
CD45
tdT+ CD45+ / CD45+

Figure S5 (Related to Figure 3). Characterization of PI16^{+} cells in a grafted artery.

(A) Schematic showing genetic lineage tracing by PI16-CreER^{T2}; Rosa26-tdTomato. (B) Representative co-immunofluorescence images of tdTomato and PI16 before (n = 6) and after (n = 6) arterial transplantation in PI16-CreER^{T2}; Rosa26-tdTomato mice; and semi-quantification of the percentage of PI16^{+} cells among tdTomato^{+} cells. Data were presented as the mean ± SEM. (C) Schematic showing the overlap of PI16 lineage cells and CD34+ cells. (D) Representative co-immunofluorescence images of tdTomato and CD34 after arterial transplantation in PI16-CreER^{T2}; Rosa26-tdTomato mice; and semi-quantification of the percentage of tdTomato^{+} cells among CD34^{+} cells. Data were presented as the mean ± SEM. (E) UMAP plots showing that the CD34 distribution in total cell clusters. (F) Pie chart showing that the proportion of PI16-positive cells in different groups. (G) Gene Ontology analysis revealing the difference of top enriched functions between PI16+ cells and PI16- cells. (H-K) Representative co-immunofluorescence images of tdTomato and CD31, α-SMA, PDGFR-α, and CD45 on cryosections from 4-week grafted arteries of PI16-CreER^{T2}; Rosa26-tdTomato mice (n = 6). Right panel: semi-quantification of the percentage of CD31, α-SMA, PDGFR-α, and CD45 cells that express tdTomato in (H-K). Data were presented as the mean ± SEM. * $p < 0.05$, ** $p < 0.01$, *** $p < 0.001$, **** $p < 0.0001$.

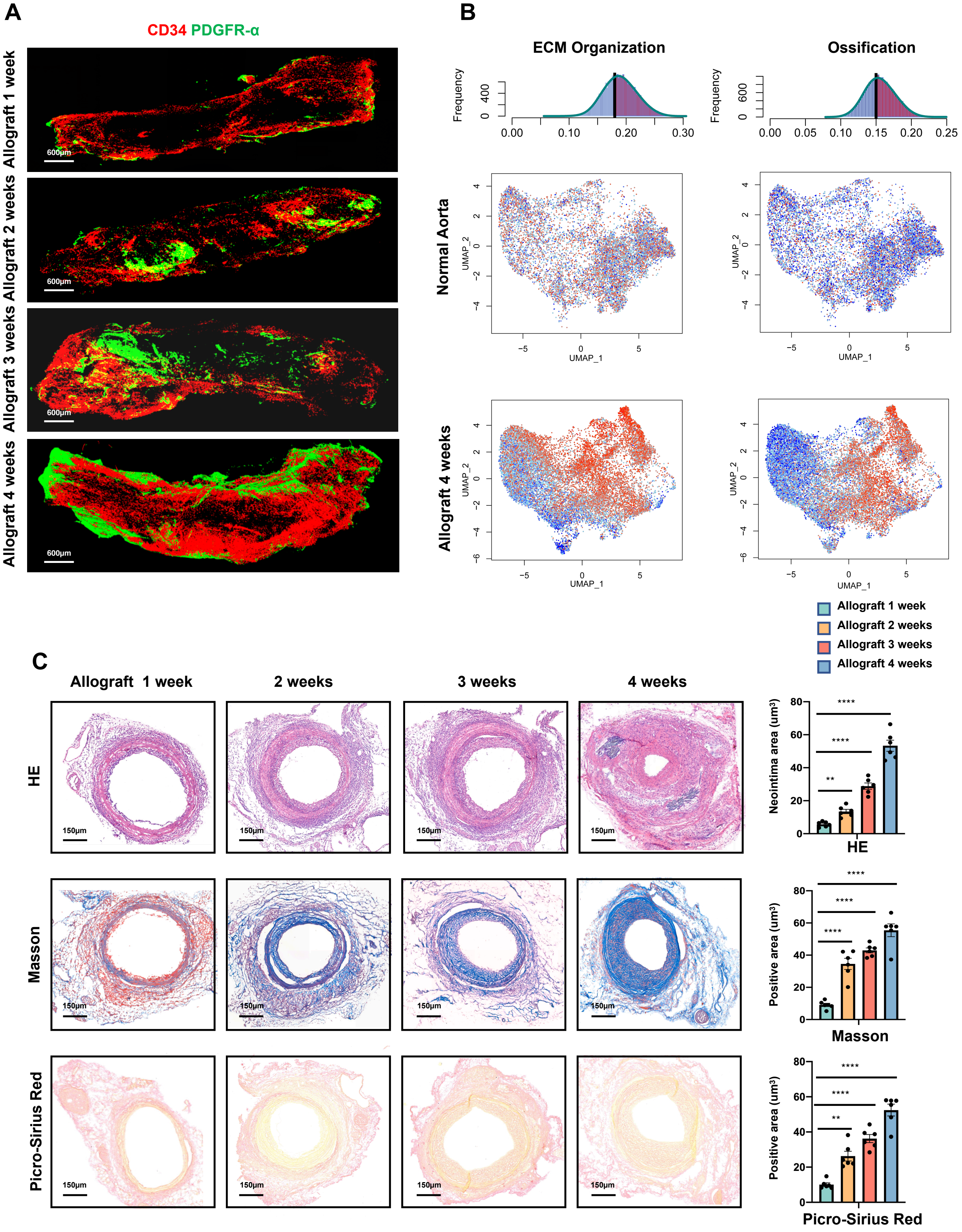
A
CD34 PDGFR-α
Allograft 1 week
Allograft 2 weeks
Allograft 3 weeks
Allograft 4 weeks
600μm
B
ECM Organization
Ossification
Frequency
Normal Aorta
Allograft 4 weeks
UMAP_1
UMAP_2
Allograft 1 week
Allograft 2 weeks
Allograft 3 weeks
Allograft 4 weeks
C
Allograft 1 week
2 weeks
3 weeks
4 weeks
HE
Masson
Picro-Sirius Red
150μm
Neointima area (um³)
Positive area (um³)
HE
Masson
Picro-Sirius Red

Figure S6 (Related to Figure 4). Alterations of adventitial remodeling in graft vasculopathy.

(A) Whole-mount Immunostaining of CD34 and PDGFR-α for grafted arteries at different points (1,2,3 and 4 weeks). n = 6 per group. Data were presented as the mean ± SEM. (B) ScRNA-sequencing analysis of ECM organization and ossification in normal thoracic aorta and 4 weeks allograft. (C) HE, Masson, and Picro-Sirius staining of grafted arteries at different points (1,2,3 and 4 weeks). n = 6 per group. Data were presented as the mean ± SEM. * $p < 0.05$, ** $p < 0.01$, *** $p < 0.001$, **** $p < 0.0001$.
