# Supplementary material for: CD34+ PI16+ fibroblast progenitors aggravate neointimal lesions of allograft arteries via CCL11/CCR3-PI3K/AKT pathway
Source: Theranostics. 2025 Jan 20;15(6):2523–43. doi: 10.7150/thno.104650 (PMC11840720; doi:10.7150/thno.104650)
Supplement: Supplementary file 3 — Supplementary figures 7-8. [file thnov15p2523s3.pdf]

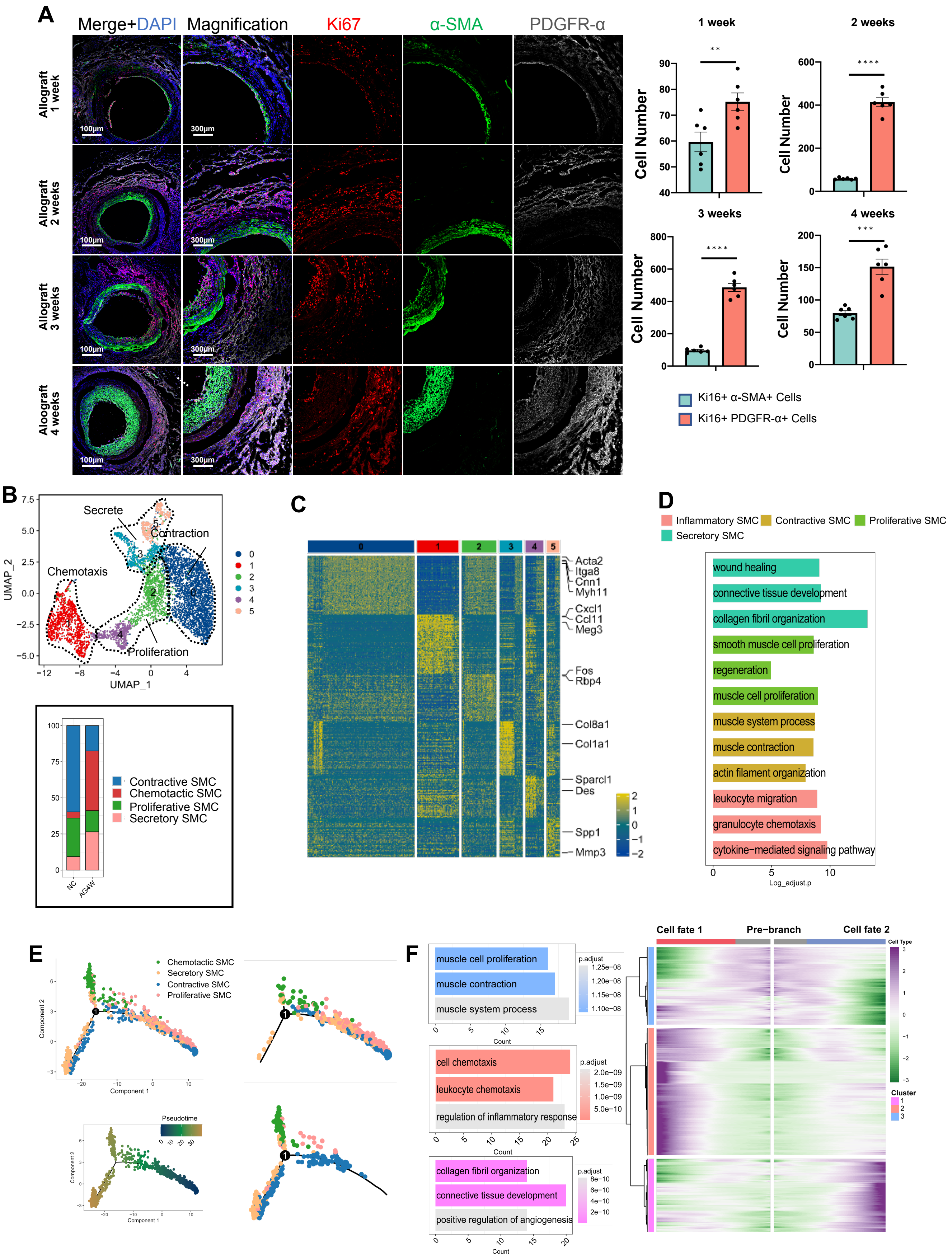
A
Merge+DAPI
Magnification
Ki67
α-SMA
PDGFR-α
Allograft 1 week
Allograft 2 weeks
Allograft 3 weeks
Alograft 4 weeks
100μm
300μm
1 week
2 weeks
3 weeks
4 weeks
Cell Number
**
****
****
***
Ki16+ α-SMA+ Cells
Ki16+ PDGFR-α+ Cells
B
Secrete
Contraction
Chemotaxis
Proliferation
UMAP_1
UMAP_2
Contractive SMC
Chemotactic SMC
Proliferative SMC
Secretory SMC
NC
AG4W
C
Acta2
Itga8
Cnn1
Myh11
Cxcl1
Ccl11
Meg3
Fos
Rbp4
Col8a1
Col1a1
Sparcl1
Des
Spp1
Mmp3
D
Inflammatory SMC
Contractive SMC
Proliferative SMC
Secretory SMC
wound healing
connective tissue development
collagen fibril organization
smooth muscle cell proliferation
regeneration
muscle cell proliferation
muscle system process
muscle contraction
actin filament organization
leukocyte migration
granulocyte chemotaxis
cytokine−mediated signaling pathway
Log_adjust.p
E
Chemotactic SMC
Secretory SMC
Contractive SMC
Proliferative SMC
Component 1
Component 2
Pseudotime
F
muscle cell proliferation
muscle contraction
muscle system process
cell chemotaxis
leukocyte chemotaxis
regulation of inflammatory response
collagen fibril organization
connective tissue development
positive regulation of angiogenesis
p.adjust
Count
Cell fate 1
Pre−branch
Cell fate 2
Cell Type
Cluster

Figure S7 (Related to Figure 6). Changes in the status of recipient-derived smooth muscle cells.

(A) Immunostaining for Ki67, PDGFR-α and α-SMA in 1, 2, 3, and 4 weeks after transplantation. n = 6 per group. Data are presented as mean ± SEM. * $p < 0.05$, ** $p < 0.01$, *** $p < 0.001$, **** $p < 0.0001$. (B) UMAP plots showing 4 groups and proportions (chemotaxis/proliferation/secrete/contraction) in the normal thoracic aorta and 4-week aortic graft. (C) Heatmap showing average scaled expression levels of top DEGs in SMCs clusters. (D) Pseudotime trajectories showing states of the SMC clusters. (E) Heatmap showing the functions and cell fate in SMCs cluster1-3. (F) Gene set enrichment analyses showing the top 10 enriched gene ontology biological processes (GOBP) in SMC clusters.

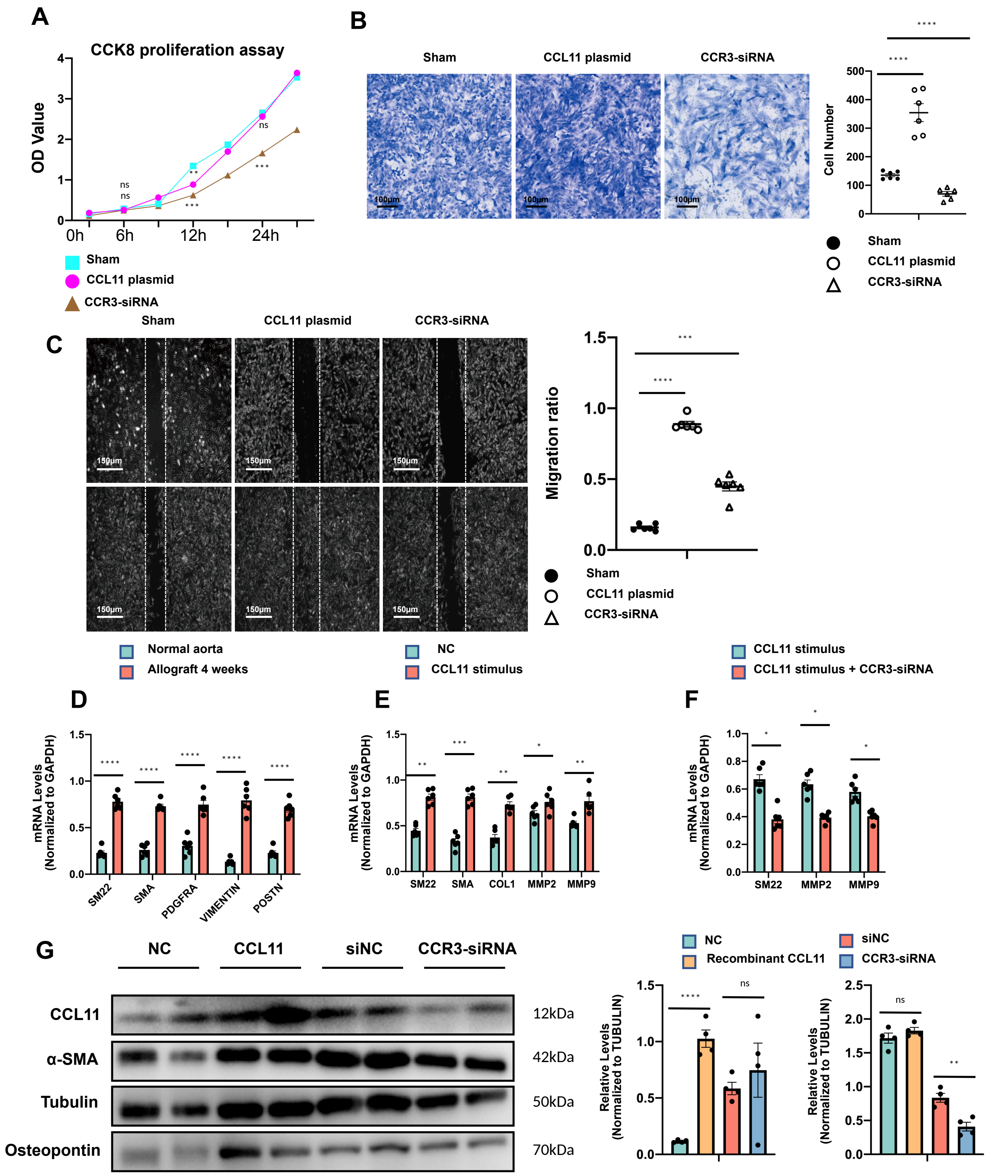

A
CCK8 proliferation assay
OD Value
0h
6h
12h
24h
Sham
CCL11 plasmid
CCR3-siRNA
B
Sham
CCL11 plasmid
CCR3-siRNA
Cell Number
C
Migration ratio
D
Normal aorta
Allograft 4 weeks
mRNA Levels (Normalized to GAPDH)
SM22
SMA
PDGFRA
VIMENTIN
POSTN
E
NC
CCL11 stimulus
COL1
MMP2
MMP9
F
CCL11 stimulus + CCR3-siRNA
G
NC
CCL11
siNC
CCR3-siRNA
CCL11
α-SMA
Tubulin
Osteopontin
12kDa
42kDa
50kDa
70kDa
Recombinant CCL11
Relative Levels (Normalized to TUBULIN)

Figure S8 (Related to Figure 7). Proliferation and migration of smooth muscle in vitro are regulated by CCL11 and CCR3.

(A) CCK8 proliferation assay of SMCs cultured with PBS, CCL11-plasmid and CCR3-siRNA for 12h (n = 6 per group). (B) Representative migration images of SMCs cultured with PBS, CCL11-plasmid and CCR3-siRNA for 12h (n = 6 per group). Right panel: quantification analysis of migration rates of SMCs among these three groups. (C) Representative scratch-healing images of SMCs cultured with PBS, CCL11-plasmid and CCR3-siRNA for 12h (n = 6 per group). Right panel: quantification analysis of scratch-healing rates of SMCs among these three groups. (D) qPCR analyses showing gene expression of these markers (SM22/α-SMA/PDGFR-α /Vimentin/Postn) in normal aorta and allograft 4 weeks (n = 6 per group). (E) qPCR analyses showing gene expression of these markers (SM22/α-SMA/CollagenaseI/MMP2/MMP9) in normal SMC group and recombinant CCL11-treating group (n = 6 per group). Cells were treated with recombinant CCL11 10ng/ul for 12h. (F) qPCR analyses showing gene expression of these markers (SM22/MMP2/MMP9) in recombinant CCL11-treating SMC group and recombinant CCL11 combined CCR3-siRNA-treating SMC group (n = 6 per group). SMCs were treated with recombinant CCL11 10ng/ul for 12h or CCR3-siRNA 10nmol for 24h. (G) Representative western blot images of these markers(CCL11/α-SMA/ Osteopontin) in SMCs treated with PBS, CCL11-plasmid and CCR3-siRNA for 12h (n = 6 per group). Right panel: quantification analysis of these markers. Data were presented as the mean ± SEM and analyzed by using an unpaired two-tailed Student’s t-test. * $p < 0.05$, ** $p < 0.01$, *** $p < 0.001$, **** $p < 0.0001$.
